# Integrated genome-wide Alu methylation and transcriptome profiling analyses reveal novel epigenetic regulatory networks associated with autism spectrum disorder

**DOI:** 10.1186/s13229-018-0213-9

**Published:** 2018-04-16

**Authors:** Thanit Saeliw, Chayanin Tangsuwansri, Surangrat Thongkorn, Weerasak Chonchaiya, Kanya Suphapeetiporn, Apiwat Mutirangura, Tewin Tencomnao, Valerie W. Hu, Tewarit Sarachana

**Affiliations:** 1 M.Sc. Program in Clinical Biochemistry and Molecular Medicine, Department of Clinical Chemistry, Faculty of Allied Health Sciences, Chulalongkorn University, Bangkok, Thailand; 2 Maximizing Thai Children’s Developmental Potential Research Unit, Department of Pediatrics, Faculty of Medicine, Chulalongkorn University and King Chulalongkorn Memorial Hospital, the Thai Red Cross Society, Bangkok, Thailand; 3 Center of Excellence for Medical Genetics, Department of Pediatrics, Faculty of Medicine, Chulalongkorn University, Bangkok, Thailand; 4 Excellence Center for Medical Genetics, King Chulalongkorn Memorial Hospital, Thai Red Cross Society, Bangkok, Thailand; 5 Center of Excellence in Molecular Genetics of Cancer and Human Diseases, Department of Anatomy, Faculty of Medicine, Chulalongkorn University, Bangkok, Thailand; 6 Age-related Inflammation and Degeneration Research Unit, Department of Clinical Chemistry, Faculty of Allied Health Sciences, Chulalongkorn University, 154 Soi Chula 12, Rama 1 Road, Wangmai, Pathumwan, Bangkok, 10330 Thailand; 7 Department of Biochemistry and Molecular Medicine, The George Washington University School of Medicine and Health Sciences, The George Washington University, Washington, DC USA

**Keywords:** Autism spectrum disorder, Alu elements, Retrotransposon, DNA methylation, Epigenetic regulation, Gene expression profiles, Subgrouping, Lymphoblastoid cell lines, Sex bias, Neuroinflammation

## Abstract

**Background:**

Alu elements are a group of repetitive elements that can influence gene expression through CpG residues and transcription factor binding. Altered gene expression and methylation profiles have been reported in various tissues and cell lines from individuals with autism spectrum disorder (ASD). However, the role of Alu elements in ASD remains unclear. We thus investigated whether Alu elements are associated with altered gene expression profiles in ASD.

**Methods:**

We obtained five blood-based gene expression profiles from the Gene Expression Omnibus database and human Alu-inserted gene lists from the TranspoGene database. Differentially expressed genes (DEGs) in ASD were identified from each study and overlapped with the human Alu-inserted genes. The biological functions and networks of Alu-inserted DEGs were then predicted by Ingenuity Pathway Analysis (IPA). A combined bisulfite restriction analysis of lymphoblastoid cell lines (LCLs) derived from 36 ASD and 20 sex- and age-matched unaffected individuals was performed to assess the global DNA methylation levels within Alu elements, and the Alu expression levels were determined by quantitative RT-PCR.

**Results:**

In ASD blood or blood-derived cells, 320 Alu-inserted genes were reproducibly differentially expressed. Biological function and pathway analysis showed that these genes were significantly associated with neurodevelopmental disorders and neurological functions involved in ASD etiology. Interestingly, estrogen receptor and androgen signaling pathways implicated in the sex bias of ASD, as well as IL-6 signaling and neuroinflammation signaling pathways, were also highlighted. Alu methylation was not significantly different between the ASD and sex- and age-matched control groups. However, significantly altered Alu methylation patterns were observed in ASD cases sub-grouped based on Autism Diagnostic Interview-Revised scores compared with matched controls. Quantitative RT-PCR analysis of Alu expression also showed significant differences between ASD subgroups. Interestingly, Alu expression was correlated with methylation status in one phenotypic ASD subgroup.

**Conclusion:**

Alu methylation and expression were altered in LCLs from ASD subgroups. Our findings highlight the association of Alu elements with gene dysregulation in ASD blood samples and warrant further investigation. Moreover, the classification of ASD individuals into subgroups based on phenotypes may be beneficial and could provide insights into the still unknown etiology and the underlying mechanisms of ASD.

**Electronic supplementary material:**

The online version of this article (10.1186/s13229-018-0213-9) contains supplementary material, which is available to authorized users.

## Background

Autism spectrum disorder (ASD) refers to a group of complex neurodevelopmental disorders that are characterized according to the Diagnostic and Statistical Manual of Mental Disorders, Fifth Edition (DSM-5) criteria by two domains: (i) behavioral impairment, including significant deficits in social interactions and communication, and (ii) restricted interests and repetitive behaviors [1]. Recent data released from the Autism and Developmental Disabilities Monitoring Network have shown a 78% increase in ASD prevalence over the past decade, and approximately 1 in 68 children in the USA have ASD [2]. This increase in ASD prevalence leads to a large economic burden, including costs for healthcare, ASD-related therapy, family-coordinated services, and special education systems [3].

A number of studies have supported the hypothesis that genetic factors are strongly associated with the etiology and susceptibility of ASD. However, abnormalities in genomic DNA are found in only 10–20% of ASD cases accumulatively, partly due to the etiological heterogeneity of ASD, which has a wide variety of different risk factors in addition to genetic factors [4]. A broad variability in clinical phenotypes of ASD individuals are thought to result from complicated interactions between genetic and environmental factors that increase ASD risk [5, 6]. Although the concordance rate among monozygotic (MZ) twins was found to range from 60% to as high as 90%, notable discordance in the ASD diagnosis within monozygotic twin pairs and significant differences in the ASD severity within ASD-concordant monozygotic twin pairs have also been observed [7, 8]. This evidence strongly suggests that environmental factors may play an important role in the etiology and/or the susceptibility of ASD.

DNA methylation is a major epigenetic regulator of gene expression and associated phenotypes. Methylation patterns in genomic DNA are generated during embryogenesis and early fetal development and are altered throughout life in response to endogenous or exogenous environmental signals. Recently, differentially methylated variants (DMVs) at specific CpG sites or differentially methylated regions (DMRs) have been investigated in ASD individuals using various types of tissues, including lymphoblastoid cell lines (LCLs) [9], whole blood [10], and brain tissue samples [11–13]. Nguyen and colleagues performed a large-scale methylation profiling analysis of LCLs derived from discordantly diagnosed (i.e., ASD and non-ASD) monozygotic twins and sibling pairs [9]. The results revealed differentially methylated genes associated with several biological functions, including gene transcription, nervous system development, and other biological mechanisms implicated in ASD. Moreover, the mRNA expression level of one ASD candidate gene (e.g., retinoic acid-related orphan receptor-alpha gene, *RORA*), which exhibits a differential methylation pattern in ASD, was also decreased in LCLs. Reduced RORA protein levels were also observed in the brain tissues of ASD individuals. This finding suggests that molecular changes in ASD peripheral cells may reflect at least some pathobiological conditions in the brain.

Several studies involving the gene expression profiling of blood or blood-derived cell lines from ASD and non-ASD subjects reported different (but somewhat overlapping) sets of differentially expressed genes (DEGs), most likely due to the heterogeneity within the ASD population. To reduce phenotypic variability and increase statistical power, Hu and Steinberg defined phenotypes within ASD based on cluster analyses of Autism Diagnostic Interview-Revised (ADI-R) scores [14]. Interestingly, gene expression profiling among the LCLs of ASD subgroups also showed differential expression [15]. This result suggested that the sub-classification of ASD patients could help identify subphenotype-specific risk factors in heterogeneous ASD populations. However, most global methylation and gene expression profiling studies have focused on protein-coding regions rather than noncoding regions that include repetitive sequences.

Alu elements are a group of repetitive sequences or mobile genetic elements with copy numbers in excess of one million in the human genome, thus contributing to almost 11% of the human genome [16]. Alu elements belong to a class of retrotransposons termed SINEs (short interspersed elements). Several reports have demonstrated that Alu elements can influence gene expression via insertion into the gene structure and can attract transcription factor binding to regulate gene expression [17]. Alu elements have many CpG residues in their sequences, and these CpG residues are common methylation sites. Methylated CpGs represent approximately 23% of all methylated residues in the human genome [18]. DNA methylation can be altered by exposure to environmental factors that can reduce Alu element methylation in human tissues [19], and there is evidence that Alu methylation plays an important role in cell proliferation and resistance to DNA damage [20]. However, the role of Alu elements and their methylation in ASD remains unclear.

**Fig. 1:**
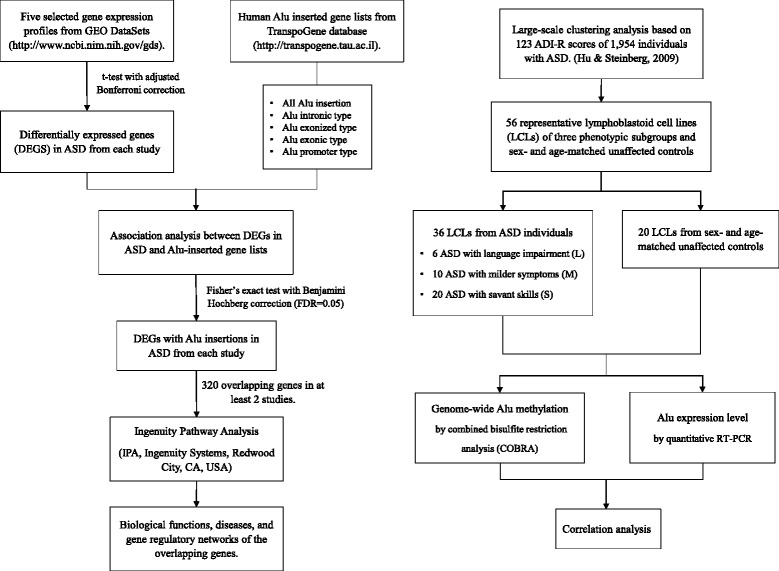
Schematic diagram of experimental workflow. Our workflow initiated with the acquisition of blood-based gene expression profiles from GEO DataSets and human Alu-inserted gene lists. Fisher’s exact test was then used to identify differentially expressed genes (DEGs) with Alu insertions. A total of 320 overlapping genes among the selected study results were used to predict biological functions, diseases, and gene regulatory networks. Fifty-six LCLs were used as a model to investigate the association between the Alu methylation status and Alu expression profiles in LCLs

In this study, we therefore aimed to investigate the association between Alu elements and altered gene expression profiles in ASD. First, publicly available gene expression data from previously published ASD transcriptome profiling studies were downloaded from the GEO DataSets database. DEGs from each ASD transcriptome study were identified, and the association between the DEGs from each study and human Alu-inserted genes were determined by Fisher’s exact test. Moreover, the DEGs with Alu insertion were identified and subsequently subjected to Ingenuity Pathway Analysis (IPA) to predict the biological functions, canonical pathways, and gene regulatory networks associated with ASD. A combined bisulfite restriction analysis (COBRA) of Alu was then performed to assess DNA methylation within Alu elements and associated CpGs, which might regulate the expression of Alu elements and Alu-inserted genes. The schematic diagram showing the experimental workflow of this study is illustrated in Fig. 1.

## Methods

### Data collection

Gene expression profiles of ASD and control individuals were obtained from the NCBI Gene Expression Omnibus database (GEO DataSets: http://www.ncbi.nlm.nih.gov/gds) using the following criteria: the included studies must be ASD studies; the studies must include blood-based gene expression profiles from microarray experiments; and the sample sizes must be greater than or equal to 40 samples. All supplementary information, including series matrix files and related platforms, was freely available in GEO DataSets [21, 22]. The details of each study are provided in Table 1.

**Table 1:** Details of the gene expression profiles obtained from GEO DataSets

GSE DataSets	Titles	Sample type	Sample information			Number of transcripts		References
			Sample matching	Sample size	Total	Cutoff filter at 70%	Available transcripts with Alu insertion	
GSE15402	Gene expression profiling differentiates autism case-controls and phenotypic variants of autism spectrum disorders	Lymphoblastoid cell lines (LCLs)	Sex (male) and age-matched	87 ASD and 29 controls	116	14,834	6118	Hu VW et al. [15]
GSE18123	Blood gene expression signatures distinguish autism spectrum disorders from controls	Whole blood	Sex (male) and age-matched	170 ASD and 115 controls	285	42,150	8575	Kong SW et al. [32]
GSE25507	Autism and increased paternal age-related changes in global levels of gene expression regulation	Peripheral blood lymphocytes	Sex (male) matched	82 ASD and 64 controls	146	43,735	9437	Alter MD et al. [30]
GSE42133	Disrupted functional networks in autism underlie early brain mal-development and provide accurate classification	Whole blood	Sex (male) matched	91 ASD and 56 controls	147	24,933	7873	Pramparo T et al. [33]
GSE6575	Gene expression in blood of children with autism spectrum disorder	Whole blood	Sex (male) and age-matched	35 ASD and 12 controls	47	43,745	9437	Gregg JP et al. [31]

Alu subfamily-inserted gene lists of Human Genome 18 (UCSC hg18, NCBI build 36.1) were downloaded from the TranspoGene database (http://transpogene.tau.ac.il), which is a publicly available database of transposed elements (TEs) located within protein-coding genes [23]. For the human Alu-inserted genes, a single gene can be inserted by multiple Alu elements with different insertion types. In this study, we obtained all genes with at least one instance of one of the four types of Alu insertions, namely, the exonic, exonized, intronic, and promoter inserts (Table 2). All human Alu subfamily consensus sequences were obtained from Repbase, which is a database of repetitive elements in eukaryotic genomes [24].

**Table 2:** Total number of the Alu-inserted gene lists from TranspoGene database

Insertion type	Alu-inserted genes (*n*)
All insertion types*	13,534
Exonized type	812
Exonic type	1593
Intronic type	13,245
Promoter type	557

We obtained the lists of human genes with at least one Alu elements from the TranspoGene database. The lists can be categorized into five types of Alu insertions: exonized, exonic, intronic, promoter, and all insertion. Note that multiple Alu elements can be inserted within a single gene with different insertion types. Therefore, the total number of Alu-inserted genes is less than the sum of the exonic, exonized, intronic, and promoter types. These lists of Alu-inserted genes were used for subsequent overlap analyses with differentially expressed genes (DEGs) in ASD

*List of genes with at least one instance of one of the four types of Alu insertions

### Identification of DEGs and association with Alu-inserted genes

The transcriptome profile from each study was analyzed separately by Multiple Experiment Viewer (MEV) [25]. All transcriptome data were filtered using a 70% cutoff, which removes transcripts for which intensity values are missing in > 30% of the samples. The available transcripts were used for identifying DEGs in ASD with two-tailed *t* tests with adjusted Bonferroni correction. All studies obtained from GEO DataSets employed ASD samples vs. sex- and/or age-matched controls in the analyses. The DEGs and non-DEGs were intersected with the human Alu-inserted gene lists, and the number of intersected genes was classified based on a crosstab 2 × 2 table into four categories, namely, DEGs with Alu insertion, DEGs without Alu insertion, non-DEGs with Alu insertion, and non-DEGs without Alu insertion. Fisher’s exact test was then used to determine whether the DEG distributions were dependent on the human Alu-inserted gene lists. Moreover, the DEGs were classified as downregulated or upregulated and used for comparison with the human Alu-inserted gene lists. These processes were repeated with each type of human Alu insertion, including intronic, exonized, exonic, and promoter type. A Fisher’s exact test *P* value with Benjamini-Hochberg correction (FDR = 0.05) of less than 0.05 was considered significant.

To identify the reproducibility of the human Alu-inserted genes that were differentially expressed in peripheral blood and cell lines derived from ASD individuals, the significant gene lists from the individual studies were used to create Venn diagrams (http://bioinformatics.psb.ugent.be/webtools/Venn). The DEGs found in at least two studies were selected to identify biological functions and gene regulatory networks that were enriched by Alu elements in ASD.

### Identification of biological functions, canonical pathways, and gene regulatory networks associated with Alu elements in ASD

IPA (QIAGEN Inc., https://www.qiagenbioinformatics.com/products/ingenuity-pathway-analysis/) is a powerful bioinformatics tool that is helpful for understanding complex “-omics” data, such as data from microarray experiments. In this study, IPA was used to identify the biological functions, diseases, canonical pathways, and gene regulatory networks of genes that were identified in at least two studies using Fisher’s exact tests with Benjamini-Hochberg correction (FDR = 0.05, *P* value < 0.05).

### Experimental models and cell culture

LCLs derived from the peripheral lymphocytes of male individuals were obtained from the Autism Genetic Resource Exchange Repository (AGRE, Los Angeles, CA, USA). Our subjects included LCLs from 36 ASD individuals and 20 sex- and age-matched unaffected controls. These individuals were previously used in large-scale clustering analysis for identification of phenotypic subgroups within ASD based on the Autism Diagnostic Interview-Revised (ADI-R) scores, as previously described in detail [14]. As a result, a total of 1954 individual ASD probands were subdivided into four phenotypic groups based on the scores of the ADI-R questionnaire. Then, we selected representative samples from the groups after excluding ASD individuals with cognitive impairment (Raven’s scores < 70), known genetic or chromosomal abnormalities (e.g., Fragile X, Rett syndrome, tuberous sclerosis, chromosome 15q11–q13 duplication), or diagnosed comorbid psychiatric disorders (e.g., bipolar disorder, obsessive compulsive disorder, severe anxiety). Impairment in spoken language was also confirmed based on low standard scores (< 80) on the Peabody Picture Vocabulary Test. Individuals born prematurely (< 35 weeks gestation) were also excluded from this study. These exclusion criteria are expected to reduce the heterogeneity of subjects to study idiopathic autism. Our LCLs represented individuals from three phenotypic groups: severe language impairment (subgroup L), milder symptoms (subgroup M), and savant skills (subgroup S). The demographic information of the LCLs used in this study is shown in Additional file 1. The LCLs were cultured according to the protocol of the Rutgers University Cell and DNA Repository, which maintains the AGRE collection of biological materials from autistic individuals and their relatives. Briefly, cells were cultured in RPMI 1640 medium supplemented with 15% fetal bovine serum and 1% penicillin/streptomycin. The cultures were split 1:2 every 3–4 days and harvested for DNA and RNA isolation 3 days after splitting, when the cultures were in the logarithmic growth phase.

### Quantitative reverse transcription-PCR analysis

Total RNA was isolated from LCLs of ASD individuals and sex- and age-matched unaffected controls using the GENEzol Reagent (Geneaid, Taiwan) according to the manufacturer’s recommended protocol. The RNA concentration was determined using a NanoDrop 1000 spectrophotometer (Thermo Scientific, USA). Quantitative reverse-transcription-PCR (RT-PCR) analysis was used to determine AluS subfamily expression in the LCLs of ASD individuals and controls. First, 5 μg of extracted RNA was treated with DNase enzyme in a 10-μl reaction (RQ1 RNase-Free DNase, Promega), and then, 2 μl of the DNase-treated RNA was reverse-transcribed to complementary DNA (cDNA) using AccuPower® RT PreMix (Bioneer, Korea) and oligo dT_18_ primer in a volume of 20 μl, according to the manufacturer’s instructions. The quantitative PCR assay was performed in triplicate using 1 μl of the cDNA in master mix reactions according to the manufacturer’s instructions (AccuPower® 2X GreenStar™ qPCR MasterMix, Bioneer, Korea). The amplification cycles consisted of an initial denaturing cycle at 95 °C for 15 min followed by 40 cycles of 45 s at 95 °C for denaturing and 45 s at 60 °C for annealing/extension. Product formation was confirmed by melting curve analysis (55 to 94 °C). The AluS transcript-specific primers were as follows: forward 5′-GTGGCTCACGCCTGTAATC-3′ and reverse 5′-GTAGAGACGGGGTTTCACCA-3′. The number of AluS transcripts was normalized to the housekeeping gene *GAPDH* whose expression was measured using the following primer sequences (forward 5′-ATGTTCGTCATGGGTGTGAA-3′ and reverse 5′-ACAGTCTTCTGGGTGGCAGT-3′), and the AluS expression level was calculated using the 2^−ΔΔCt^ method.

### Determination of the Alu methylation levels and patterns in LCLs

**Fig. 2:**
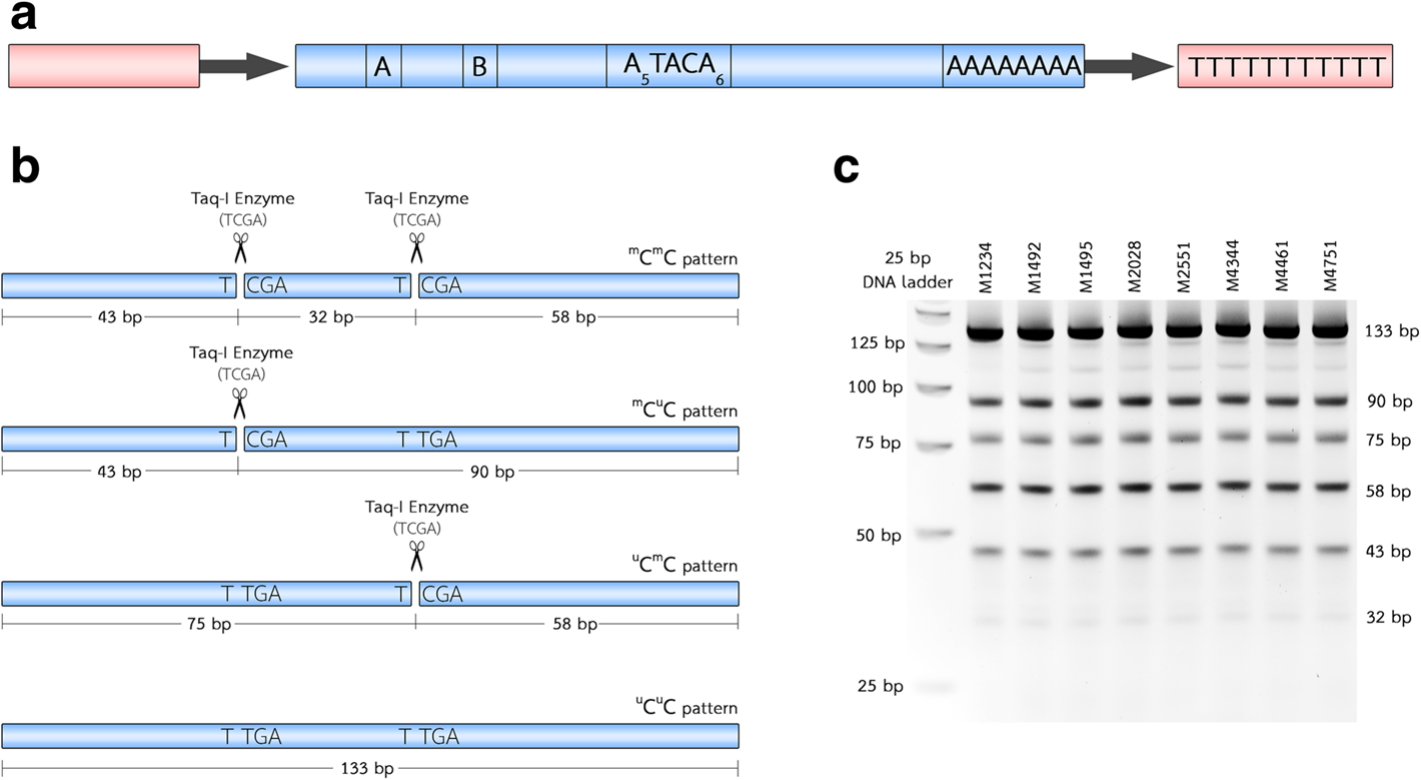
Alu element structure and illustration of COBRA for determining AluS methylation levels and patterns. **a** Alu elements are approximately 300 bp in length and have a dimeric structure that is separated by an A-rich region (A_5_TACA_6_) and ends with a poly-A tail. The left half of the Alu contains the A and B boxes, which are internal promoters for RNA polymerase III. **b** Illustration of the COBRA method designed to assess methylation of two CpGs at the internal promoter of AluS subfamilies. The four different methylation patterns of AluS were calculated from the percentages of differently digested products of 133, 75, 58, 43, and 32 bp. **c** Representative gel image from the COBRA for AluS subfamilies

Genomic DNA was isolated from LCLs of ASD individuals and sex-/age-matched unaffected controls using the GENEzol Reagent (Geneaid, Taiwan) according to the manufacturer’s recommended protocol. The DNA concentration was determined using a NanoDrop 1000 spectrophotometer (Thermo Scientific, USA). COBRA is designed to determine methylation levels and patterns of two CpG loci within AluS subfamilies with the highest number of copies and CpG loci in the human genome (Fig. 2a). Briefly, 1 μg of genomic DNA from each sample was treated with sodium bisulfite using EZ DNA Methylation-Gold™ Kit (Zymo, Irving, CA, USA). The bisulfite-treated DNA was subjected to 45 cycles of PCR (Hot Start Taq DNA polymerase, QIAGEN, USA) with two specific primers for AluS subfamilies, AluS-F (5′-GGRGRGGTGGTTTARGTTTGTAA-3′) and AluS-R (5′-CTAACTTTTTATATTTTTAATAAAAACRAAATTTCACCA-3′), at 53 °C for annealing. Then, the AluS amplicons were digested with TaqI restriction enzyme (Thermo Scientific, USA) in TaqI buffer and incubated at 65 °C overnight. Finally, the digested products were electrophoresed on an 8% non-denaturing polyacrylamide gel, and band intensities were determined for assessment of the AluS methylation levels and patterns as described previously [26].

Upon gel electrophoresis, the digested Alu-amplicons were resolved into six fragments of 133, 90, 75, 58, 43, and 32 bp, which represented different methylation states (Fig. 2b, c), including the ^u^C^u^C methylation state (represented by the 133-bp fragment). The ^m^C^u^C methylation state was represented by the 90-bp fragment. The ^u^C^m^C methylation state was represented by the 75- and 58-bp fragments. The ^m^C^m^C methylation state was represented by the 43- and 32-bp fragments. The calculation for percent AluS methylation was performed as follows. First, the percentage of band intensity was divided by the length (bp) of each DNA fragment: %133/133 = A, %58/58 = B, %75/75 = C, %90/90 = D, %43/43 = E, and %32/32 = F. The percentages of Alu methylation levels and patterns were then calculated using the following formulas: percentage methylated loci (%^m^C) = 100 × (E + B)/(2A + E + B + C + D), percentage of hypermethylated pattern (%^m^C^m^C) = 100 × F/(A + C + D + F), percentage of partially methylated pattern (%^u^C^m^C) = 100 × C/(A + C + D + F), percentage of partially methylated pattern (%^m^C^u^C) = 100 × D/(A + C + D + F), and percentage of partially hypomethylated pattern (%^u^C^u^C) = 100 × A/(A + C + D + F).

### Statistical analyses

DEGs were determined using two-tailed *t* tests with adjusted Bonferroni correction, and adjusted *P* values less than 0.05 were considered significant. Fisher’s exact test with Benjamini-Hochberg (FDR = 0.05) correction was used to investigate the association between the DEG lists and the human Alu-inserted gene lists. *P* values less than 0.05 were considered significant. Pathway and function analyses were performed with IPA using Fisher’s exact test with Benjamini-Hochberg correction for multiple testing (FDR = 0.05); *P* values less than 0.05 were considered significant. Two-tailed *t* tests with Benjamini-Hochberg correction (FDR = 0.05) were used to analyze the differences in AluS methylation and expression level; the adjusted *P* value threshold was 0.05.

## Results

### Genes containing Alu elements are differentially expressed in ASD blood based on the integration of data from multiple studies

We hypothesized that Alu elements are associated with altered gene expression in the peripheral blood and blood-derived cell lines of ASD individuals and provided CpG sites for DNA methylation within Alu-inserted genes. To test this hypothesis, we obtained five transcriptome profiles from ASD studies available in GEO DataSets and identified a list of DEGs from each study. We then overlapped these DEG lists with the human Alu-inserted genes using Fisher’s exact test; the results are shown in Table 3. These results showed that the DEGs in the peripheral blood of ASD individuals were significantly associated with the Alu-inserted gene lists. For the group of all Alu insertion types, the lists of DEGs (up- and downregulated genes) were significantly associated with Alu elements. These lists consist of 388, 869, 1001, and 1492 DEGs from the GSE6575, GSE25507, GSE42133, and GSE18123 studies, respectively. We subsequently identified the up- and downregulated DEGs and assessed their overlap with the “all Alu insertion” gene list type; the results showed a strong association for downregulated genes (adjusted *P* value < 0.0005). However, the “all Alu insertion” type was only weakly associated with upregulated genes (adjusted *P* value = 0.015) in the GSE42133 study and showed a weaker association in the other studies. DEGs were also compared with the other types of Alu insertion gene lists, including the exonic, exonized, intronic, and promoter types. The results are shown in Table 3. These results showed that the DEGs were more strongly associated with the Alu intronic lists than with the other types of Alu insertions. Intronic Alu elements were also strongly associated with the downregulated gene lists. Moreover, because the GSE15402 study has also reduced the heterogeneity of ASD by subgrouping ASD individuals based on their clinical phenotypes using supervised and unsupervised clustering analyses of ADI-R scores, we further investigated whether Alu insertions were associated with DEGs in each ASD subgroup. Interestingly, we found that genes with Alu insertion were significantly associated with DEGs in ASD subgroups but not when all ASD cases were combined (Additional file 2).

**Table 3:** Association analyses between the differentially expressed genes (DEGs) in ASD and the human Alu-inserted gene lists

Insertion type	Comparison	GEO datasets	All differentially expressed genes		Upregulated genes		Downregulated genes	
			*P* value	Genes (*n*)	*P* value	Genes (*n*)	*P* value	Genes (*n*)
All insertion	ASD vs. control	GSE15402	0.799	215	0.926	100	0.799	116
		GSE18123	< 0.00005	1492	1.000	255	< 0.00005	1245
		GSE25507	0.012	869	0.256	522	< 0.00005	355
		GSE42133	< 0.00005	1001	0.015	387	< 0.00005	624
		GSE6575	< 0.00005	388	0.955	123	< 0.00005	266
Intronic	ASD vs. control	GSE15402	0.784	212	0.926	99	0.784	114
		GSE18123	< 0.00005	1476	0.970	252	< 0.00005	1231
		GSE25507	0.007	860	0.322	516	< 0.00005	352
		GSE42133	< 0.00005	985	0.012	382	< 0.00005	613
		GSE6575	< 0.00005	382	0.882	119	< 0.00005	264
Exonized	ASD vs. control	GSE15402	1.000	15	0.933	6	0.933	9
		GSE18123	0.009	102	0.926	14	0.003	88
		GSE25507	0.825	45	0.008	17	0.012	28
		GSE42133	0.008	75	0.306	27	0.015	49
		GSE6575	0.006	33	1.000	7	< 0.00005	26
Exonic	ASD vs. control	GSE15402	0.450	19	0.904	10	0.426	9
		GSE18123	0.001	177	0.306	21	< 0.00005	158
		GSE25507	0.426	78	0.136	46	0.662	33
		GSE42133	< 0.00005	148	0.799	43	< 0.00005	106
		GSE6575	0.034	48	0.715	16	0.013	33
Promoter	ASD vs. control	GSE15402	0.933	6	0.436	1	0.799	5
		GSE18123	0.240	37	0.898	8	0.268	29
		GSE25507	0.135	22	0.033	11	0.937	11
		GSE42133	0.831	35	0.447	19	0.255	16
		GSE6575	0.466	16	0.716	3	0.123	13

The list of DEGs from each gene expression profiling study was overlapped with the lists of Alu-inserted genes. Alu-inserted genes were categorized into five types of Alu insertions which included exonized, exonic, intronic, promoter, and combined insertion types. Fisher’s exact test with Benjamini-Hochberg correction (FDR = 0.05) was used to determine the association the DEGs and Alu-inserted genes, and *P* values of less than 0.05 were considered significant. The number of DEGs and adjusted *P* values are shown

**Fig. 3:**
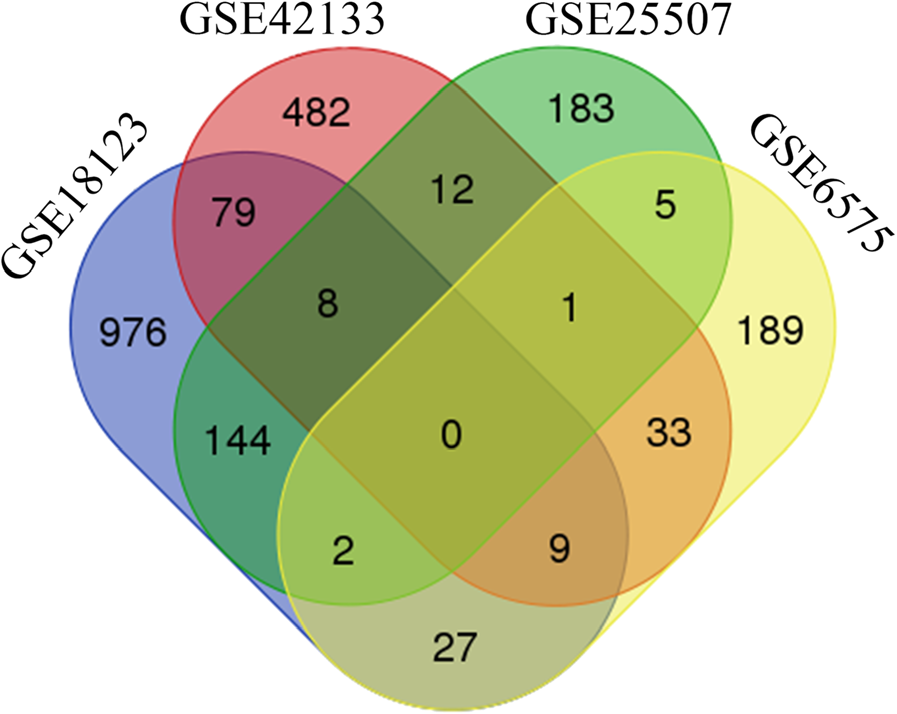
Venn diagram of genes containing Alu that are differentially expressed in ASD. The significant DEGs with Alu insertions from each study based on Fisher’s exact test overlapped. The diagram shows the reproducibility of Alu-inserted genes that were differentially expressed in peripheral blood and blood-derived cell lines from ASD individuals. A total of 320 genes were selected to identify biological functions and gene regulatory networks through an Ingenuity Pathway Analysis (IPA)

We subsequently selected the lists of downregulated genes that were significantly associated with all Alu insertion lists from each study. These lists were used to identify overlapping genes among the selected studies using Venn diagram analysis (Fig. 3). Interestingly, the diagram revealed the reproducibility of Alu-inserted genes that were differentially expressed in ASD whole blood/blood cells. Significant genes that were identified in at least two studies (320 overlapping genes; see Additional file 3) were selected to identify gene regulatory networks, diseases, and biological functions that were associated with Alu elements in ASD.

### Biological functions, canonical pathways, and gene regulatory networks of dysregulated genes containing Alu elements are associated with neurological functions and neurodevelopmental disorders

IPA was used to predict the diseases, biological functions, canonical pathways, and gene regulatory networks that were associated with Alu elements in ASD. The results showed that the overlapping genes (320 genes) were significantly associated with neurological diseases and nervous system development and function (adjusted Fisher’s exact test, *P* value < 0.05) (Table 4). Interestingly, 21 overlapping genes were associated with autism or intellectual disability. Eighteen and 20 of the overlapping genes were also associated with mental retardation and cognitive impairment, respectively, which were classified as neurodevelopmental disorders comorbid with ASD. The IPA results also revealed significant canonical pathways associated with the overlapping genes (Table 5), including neurotrophin/TRK signaling, ERK/MAPK signaling, axonal guidance signaling, CREB signaling in neurons, estrogen receptor signaling, androgen signaling, IL-6 signaling, and neuroinflammation signaling, all of which have been associated with ASD.

**Table 4:** Diseases and biological functions associated with reproducible DEGs with Alu insertion predicted by the Ingenuity Pathway Analysis (IPA)

Disease or function annotation	Benjamini-Hochberg *P* value	No. of genes	Gene symbol
Autism or intellectual disability	2.19E−04	21	ABCB1, ADNP, ANKRD11, ARID1A, ATP6V1A, CAMTA1, CASP2, CDC42, CHD4, COL4A3BP, CREBBP, GNB1, OPA1, PTEN, SLC35A3, SMARCA2, SON, TRIO, UBE3A, YY1, ZMYND11
Neuromuscular disease	5.70E−04	34	ABCB1, ADAM10, ALCAM, ANKRD11, ATP2A2, ATP6V1A, ATXN1, CANX, CASP2, CFLAR, GSK3B, HBP1, HMGCR, HSPA5, IFNAR2, IL7R, KIF1B, LDLR, MAP2K4, MBP, MBTPS1, NOTCH2, OSBPL8, PPP3CB, PTPRC, PTPRE, RUNX3, SSX2IP, TLR2, TOMM20, TRIO, USP13, WNK1, XRCC6
Synthesis of reactive oxygen species	7.52E−04	14	CANX, CDC42, CYBB, ETS1, FCER1A, HGF, ITGB1, MAP2K4, MAPK1, PIK3CG, PTEN, SHC1, TLR2, TXNRD1
Disorder of basal ganglia	9.21E−04	29	ABCB1, ANKRD11, ATP2A2, ATP6V1A, ATXN1, CA2, CASP2, CFLAR, GSK3B, HBP1, HMGCR, HSPA5, KIF1B, LDLR, MAP2K4, MBP, MBTPS1, NOTCH2, OSBPL8, PTPRE, RUNX3, SAMHD1, SSX2IP, TOMM20, TRIO, USP13, WNK1, XPR1, XRCC6
Dyskinesia	9.44E−04	23	ABCB1, ANKRD11, ATP2A2, ATP6V1A, ATXN1, CASP2, CFLAR, HBP1, HMGCR, HSPA5, LDLR, MAP2K4, MBTPS1, NOTCH2, OSBPL8, PTPRE, RUNX3, SSX2IP, TOMM20, TRIO, USP13, WNK1, XRCC6
Mental retardation	1.13E−03	18	ADNP, ANKRD11, ARID1A, ATP6V1A, CAMTA1, CASP2, CDC42, CHD4, COL4A3BP, CREBBP, GNB1, OPA1, SLC35A3, SMARCA2, SON, TRIO, YY1, ZMYND11
Brain lesion	1.36E−03	33	ANKRD11, ANXA7, APC, ARCN1, ARID1A, ATP6V1A, CA2, CBL, CREBBP, CTBP2, DICER1, DOCK5, EHD4, HGF, HMGCR, IRS2, LDLR, LYST, NCOA1, NF1, PABPC1, PIK3R1, PRKCSH, PTEN, PTPN11, SAP130, SON, TBK1, TOP1, TRIM33, TRIP11, TRRAP, ZCCHC6
Cognitive impairment	1.44E−03	20	ADNP, ANKRD11, ARID1A, ATP6V1A, CA2, CAMTA1, CASP2, CDC42, CHD4, COL4A3BP, CREBBP, GNB1, HMGCR, OPA1, SLC35A3, SMARCA2, SON, TRIO, YY1, ZMYND11
Dementia	1.62E−03	27	ADAM10, APLP2, ATXN1, CA2, CANX, CASP2, DICER1, GSK3B, HMGCR, HSPA5, LDLR, LIMS1, NFE2L2, OPA1, PIK3R1, PTEN, PTPRE, RUNX3, SLC6A6, SMPD1, SPG21, SRPK2, TBK1, TFCP2, TRIO, UBQLN1, WDR7

Neurological diseases and functions are significantly associated with 320 overlapping genes that were identified in multiple studies. *P* values calculated by Fisher’s exact test with Benjamini-Hochberg correction (FDR = 0.05) and the number of genes for each function are shown

**Table 5:** Canonical pathways associated with reproducible DEGs with Alu insertion predicted by the Ingenuity Pathway Analysis (IPA)

Ingenuity canonical pathways	Benjamini-Hochberg *P* value	Gene symbol
ILK signaling	6.39E−08	PPP2R5E, GSK3B, CDC42, PTEN, IRS2, CREB1, PIK3R4, RHOQ, PIK3R1, MYH9, PIK3CG, PTPN11, MAP2K4, ITGB1, MAPK1, CREBBP, LIMS1, FNBP1, NACA
Neurotrophin/TRK signaling	3.20E−07	CDC42, MAP3K5, IRS2, CREB1, PTPN11, MAP2K4, PIK3R4, MAPK1, CREBBP, PIK3R1, SHC1, PIK3CG
NGF signaling	5.11E−07	CDC42, MAP3K5, IRS2, CREB1, PIK3R4, SMPD1, PIK3R1, PIK3CG, PTPN11, MAP2K4, TRIO, MAPK1, CREBBP, SHC1
Reelin signaling in neurons	1.02E−06	GSK3B, ITGAL, IRS2, FYN, PTPN11, MAP2K4, ITGB1, PIK3R4, PIK3R1, YES1, LYN, PIK3CG
HGF signaling	1.24E−06	CDC42, MAP3K5, IRS2, PIK3R4, ELF2, PIK3R1, PIK3CG, PTPN11, MAP2K4, ITGB1, MAPK1, ETS1, HGF
ERK/MAPK signaling	2.73E−06	PPP2R5E, PAK2, IRS2, CREB1, PIK3R4, ELF2, PRKAG2, PIK3R1, PIK3CG, FYN, PTPN11, ITGB1, MAPK1, CREBBP, ETS1, SHC1
Insulin receptor signaling	5.09E−06	GSK3B, PTEN, IRS2, PIK3R4, PRKAG2, RHOQ, PIK3R1, CBL, PIK3CG, FYN, PTPN11, MAPK1, SHC1
Axonal guidance signaling	2.47E−05	SEMA4D, GSK3B, CDC42, PAK2, IRS2, PIK3R4, PLXNC1, GNB1, PPP3CB, PRKAG2, ADAM10, PIK3R1, RASSF5, PIK3CG, GNAI2, FYN, PPP3R1, PTPN11, ITGB1, MAPK1, PLCL2, SHC1
IL-6 signaling	4.05E−05	ABCB1, MAP4K4, IRS2, PTPN11, MAP2K4, PIK3R4, MAPK1, PIK3R1, SHC1, IL6ST, PIK3CG
Neuroinflammation signaling pathway	4.51E−05	GSK3B, IRS2, CREB1, PIK3R4, PPP3CB, PIK3R1, TBK1, PIK3CG, CFLAR, PPP3R1, PTPN11, MAP2K4, MAPK1, CREBBP, CYBB, TLR2, NFE2L2
Glucocorticoid receptor signaling	4.78E−05	IRS2, CREB1, NCOA1, PIK3R4, SMARCA2, PPP3CB, PRKAG2, PIK3R1, PIK3CG, TAF4, PPP3R1, PTPN11, MAP2K4, HSPA5, MAPK1, CREBBP, ARID1A, SHC1
PI3K/AKT signaling	1.22E−04	PPP2R5E, GSK3B, MAP3K5, PTEN, ITGB1, MAPK1, LIMS1, PIK3R1, SHC1, PIK3CG
CREB signaling in neurons	1.49E−04	IRS2, CREB1, PIK3R4, GNB1, PRKAG2, PIK3R1, PIK3CG, GNAI2, PTPN11, MAPK1, CREBBP, PLCL2, SHC1
Synaptic long-term potentiation	6.29E−03	PPP3R1, CREB1, PPP3CB, MAPK1, CREBBP, PRKAG2, PLCL2
Estrogen receptor signaling	9.05E−03	TAF4, NCOA1, CTBP2, MAPK1, CREBBP, TRRAP, SHC1
Androgen signaling	1.19E−02	GNAI2, NCOA1, GNB1, MAPK1, CREBBP, PRKAG2, SHC1

Canonical pathways are significantly associated with 320 overlapping genes that were identified in multiple studies. *P* values calculated by Fisher’s exact test with Benjamini-Hochberg correction (FDR = 0.05) for each function are shown

**Fig. 4:**
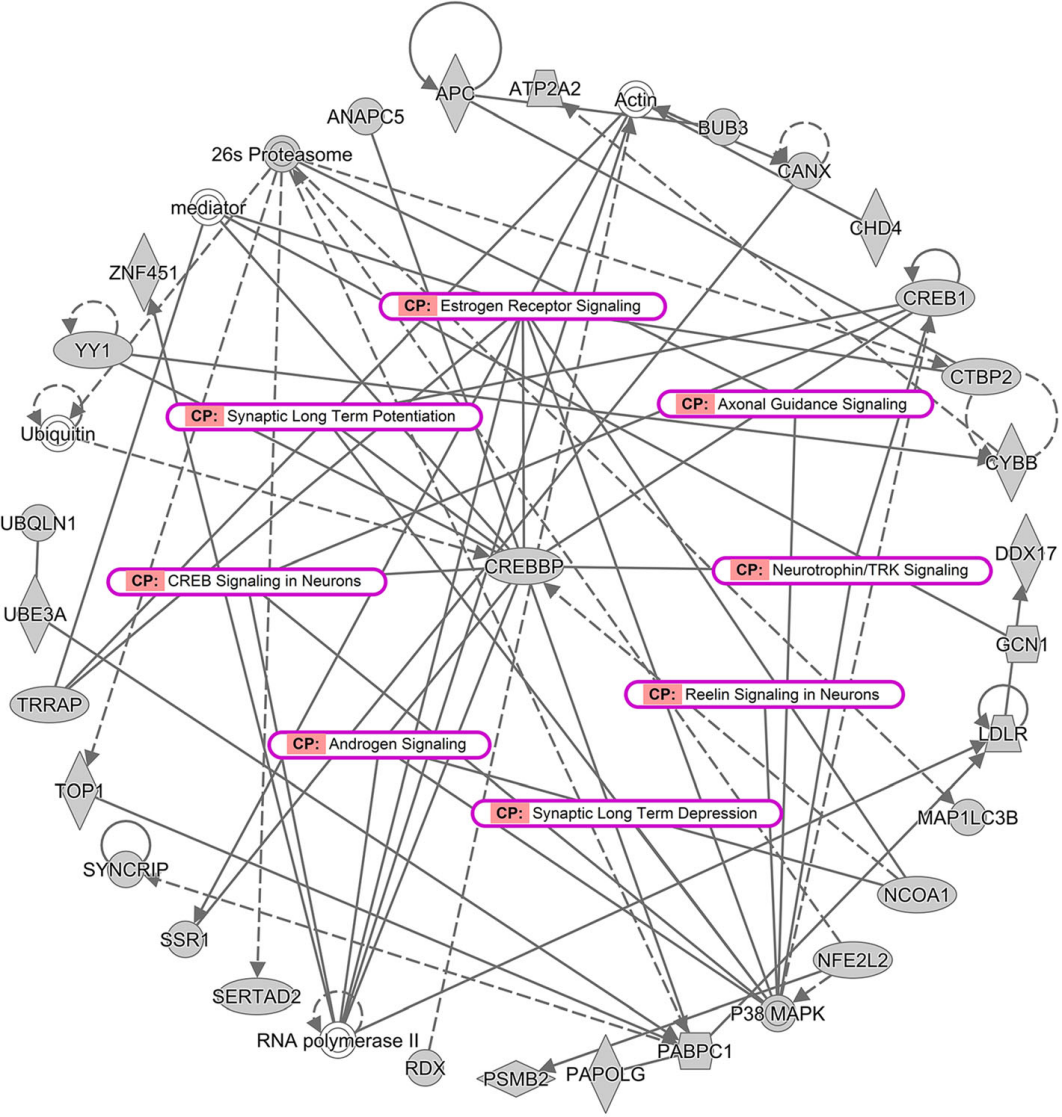
Predicted gene regulatory network of the overlapping genes associated with neurological disease. This network revealed interactions or relationships among the overlapping molecules (gray background) and with other molecules from the IPA database (white background) that play a role in several mechanisms associated with neurological disease and estrogen receptor and androgen signaling, which is known to be associated with sex bias in ASD (labeled pink)

Gene regulatory networks are sets of genes that interact to control a specific cell function, including cell differentiation, metabolism, cell cycle, and response to environmental cues. A representative gene network associated with neurological disease (Fig. 4) revealed the interaction of overlapping genes with additional molecules from the IPA database involved in estrogen receptor and androgen signaling, axonal guidance signaling, CREB signaling in neurons, and neurotrophin/TRK signaling. This network suggested that some ASD-related biological pathways are associated with Alu-inserted DEGs in the blood of ASD individuals.

### AluS methylation levels and patterns in LCLs

To determine the levels of DNA methylation within Alu elements, which contributes to a large proportion of the CpG sites in the human genome, we used LCLs as a model and conducted a COBRA. Specifically, a COBRA of AluS subfamilies was performed to determine the percentages of AluS methylation levels (^m^C) and patterns (^m^C^m^C, ^m^C^u^C, ^u^C^m^C, ^u^C^u^C) in LCLs, and the results showed that comparisons of the AluS methylation levels and patterns between the ASD (*n* = 36) and sex- and age-matched unaffected control (*n* = 20) groups were not significantly different (Table 6). The levels and patterns of Alu methylation in individual LCLs are shown in Additional file 4. Due to heterogeneity within ASD individuals, our LCLs were categorized into three subgroups, namely, savant (S), mild (M), and language-impaired (L), based on the ADI-R interview scores according to Hu and Steinberg [14]. In addition, Hu and colleagues reported that the gene expression profiles of LCLs were significantly different among these ASD subgroups [15].

**Table 6:** COBRA-derived percentages of AluS methylation and patterns in LCLs from ASD individuals and sex- and age-matched controls

Comparison	Sample groups	Age (mean ± SD)	Percentages of AluS methylation patterns				
			%^m^C	%^m^C^m^C	%^u^C^m^C	%^m^C^u^C	%^u^C^u^C
ASD vs. control (sex- and age-matched)	Control (*n* = 20)	15 ± 6.97	37.98 ± 1.36	25.70 ± 2.36	18.71 ± 1.15	21.76 ± 1.03	33.83 ± 1.69
	ASD (*n* = 36)	13.4 ± 4.55	37.86 ± 2.07	25.92 ± 2.69	18.72 ± 1.66	21.22 ± 1.76	34.13 ± 3.00
	*P* value		0.866	0.866	0.974	0.315	0.845
Subgroup M vs. control (sex- and age-matched)	Control (*n* = 10)	12.1 ± 3.81	38.07 ± 1.52	25.57 ± 2.31	18.76 ± 0.87	22.06 ± 0.69	33.62 ± 1.85
	ASD subgroup M (*n* = 10)	12.1 ± 3.73	39.03 ± 1.16	25.17 ± 1.15	20.06 ± 0.92	22.99 ± 0.82	31.77 ± 1.76
	*P* value		0.293	0.845	*0.043*	0.089	0.168
Subgroup L vs. control (sex- and age-matched)	Control (*n* = 6)	13.7 ± 1.64	37.84 ± 1.51	24.88 ± 2.94	19.10 ± 1.60	22.25 ± 1.03	33.77 ± 1.84
	ASD subgroup L (*n* = 6)	13.5 ± 1.97	36.024 ± 1.43	23.37 ± 2.63	19.32 ± 1.69	20.93 ± 2.52	36.38 ± 2.29
	*P* value		0.168	0.569	0.866	0.502	0.168
Subgroup S vs. control (sex- and age-matched)	Control (*n* = 20)	15 ± 6.97	37.98 ± 1.36	25.70 ± 2.36	18.71 ± 1.15	21.76 ± 1.03	33.83 ± 1.69
	ASD subgroup S (*n* = 20)	15 ± 5.41	37.83 ± 2.23	27.06 ± 2.67	17.88 ± 1.44	20.43 ± 1.17	34.64 ± 3.00
	*P* value		0.866	0.242	0.168	*0.010*	0.502

The percentages of AluS methylation patterns were determined based on four patterns: the hypermethylated pattern (^m^C^m^C), two partially methylated patterns (^m^C^u^C, ^u^C^m^C), and the hypomethylated pattern (^u^C^u^C). Comparisons of the methylation status between ASD and sex- and age-matched unaffected control groups and between ASD phenotypic subgroups and the matched unaffected controls were also performed. Statistically significant *P* values < 0.05 with Benjamini-Hochberg correction (FDR = 0.05) are shown in italics

**Fig. 5:**
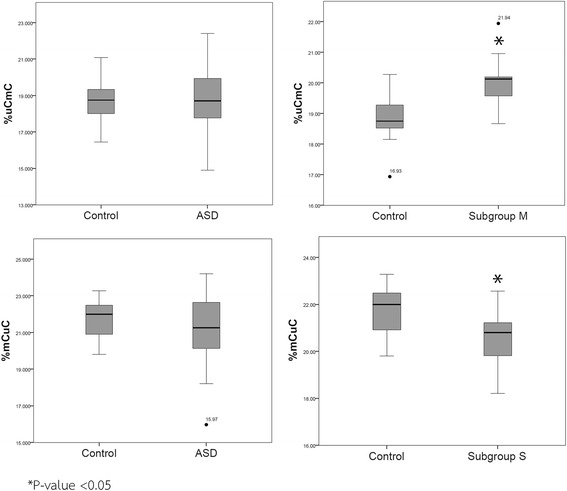
Box plot of the Alu methylation patterns in the LCLs of ASD subgroup M. In ASD subgroup M, the percentage of the partially methylated pattern ^u^C^m^C (20.06% ± 0.92%) was significantly increased. In ASD subgroup S, the partially methylated pattern ^m^C^u^C was significantly decreased. *adjusted *P* value < 0.05

We then compared the AluS methylation levels and patterns between each ASD subgroup and the sex- and age-matched groups. Interestingly, the results showed significant AluS methylation patterns associated with specific ASD subgroups. The percentage of the partially methylated pattern ^u^C^m^C (20.06% ± 0.92%, adjusted *P* value = 0.043) was significantly increased in ASD subgroup M (Table 6, Fig. 5). In addition, the percentage of the partially methylated pattern ^m^C^u^C (20.43% ± 1.17%, adjusted *P* value = 0.010) was significantly decreased in ASD subgroup S compared with sex- and age-matched controls. There were no significant patterns of AluS methylation in ASD subgroup L. These findings suggested that methylation of Alu elements might play a role in AluS expression and/or transcriptional profile of some ASD subgroups but not all ASD individuals.

### Quantitative reverse transcription-PCR analysis of the AluS expression levels in 56 LCLs

To understand the influence of DNA methylation within AluS elements on AluS expression levels, a quantitative RT-PCR analysis was performed using the LCLs of ASD and sex- and age-matched unaffected control groups. The results showed no significant differences in AluS expression between the ASD and the matched control groups (fold change (FC) = 1.75, adjusted *P* value 0.316, Table 7), which was similar to the results obtained from the AluS methylation level and pattern analyses. However, comparisons of the AluS expression levels between the ASD phenotypic subgroups and sex- and age-matched control groups revealed that these were significantly different. Specifically, the AluS expression levels were significantly decreased in ASD subgroup L (FC = 0.29, adjusted *P* value = 0.032) and significantly increased in ASD subgroup S (FC = 3.68, adjusted *P* value = 0.038). However, in ASD subgroup M, the AluS expression level was not significantly different.

**Table 7:** Quantitative RT-PCR analyses of AluS expression levels in the LCLs of ASD and control groups

Group	Fold change (FC)	Log_2_ (FC)	*P* value
ASD vs. control	1.75	0.81	0.316
Subgroup M vs. control	1.05	0.06	0.953
Subgroup L vs. control	0.29	− 1.77	0.032
Subgroup S vs. control	3.68	1.88	0.038

The levels of Alu transcripts were normalized to the housekeeping gene *GAPDH*. The AluS expression levels were calculated using the 2^−ΔΔCt^ method, and differences with a *P* value < 0.05, as determined by two-tailed *t* tests with Benjamini-Hochberg correction, were considered significant

### Correlation analysis between AluS methylation levels and AluS expression levels

**Fig. 6:**
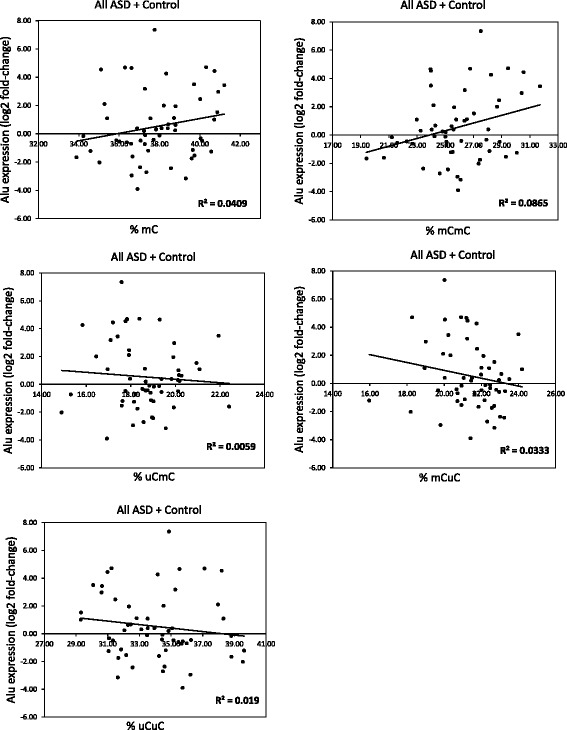
Correlation analysis between AluS methylation and expression level for all LCL samples. The AluS expression for each LCL was normalized with the average *GAPDH* dCt of the control group. The Alu expression levels were then calculated using the 2^−ΔΔCt^ method

**Fig. 7:**
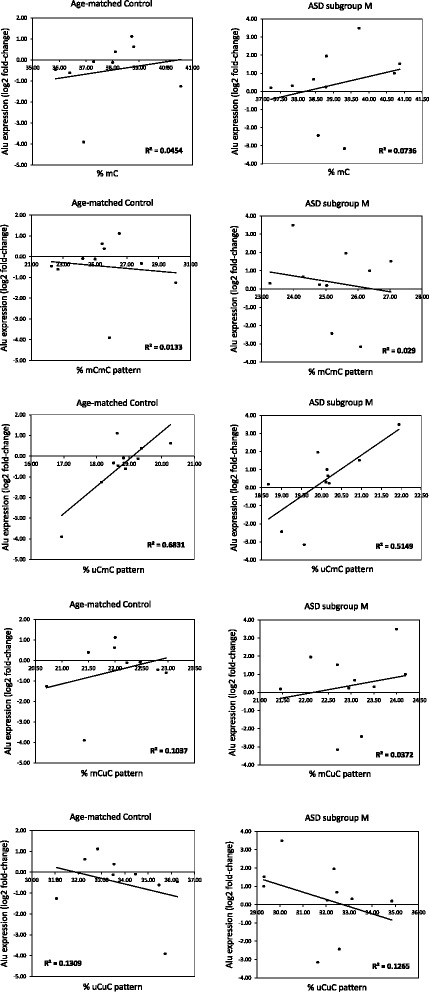
Correlation analysis between AluS methylation and expression levels in ASD subgroup M and sex- and age-matched controls. The AluS expression of each LCL was normalized to the average *GAPDH* dCt of the control group. The Alu expression levels were then calculated using the 2^−ΔΔCt^ method

To demonstrate that AluS methylation regulates the expression of AluS elements, we correlated the DNA methylation and expression levels of AluS subfamilies in the LCLs from ASD individuals and sex- and age-matched controls. The correlation analyses revealed non-linear relationships between DNA methylation and the expression of AluS subfamilies in all groups (ASD + control, Fig. 6). However, when analyzed within the ASD subgroups, we found that the partially methylated pattern ^u^C^m^C showed a moderate positive relationship with AluS expression in ASD subgroup M (coefficient *r* = 0.5149, Fig. 7), whereas other AluS methylation patterns did not correlate with ASD subgroup M. Similarly, DNA methylation and expression of AluS subfamilies were not correlated in ASD subgroups L and S (Additional files 5 and 6). These findings suggested that the partially methylated pattern ^u^C^m^C might regulate the expression of AluS elements in ASD subgroup M.

## Discussion

The mechanisms through which human Alu elements are involved in ASD are unclear. In a previous study, Mbarek et al. found a polymorphism of an Alu element in the blood of ASD individuals with more severe clinical symptoms [27]. This allele is located in intron 27b of the neurofibromatosis type 1 (*NF1*) gene, which is reported to be an ASD risk gene. However, substantially more information was needed to conclude that Alu elements play important roles in ASD. Our findings provide preliminary information regarding the association between DEGs and Alu elements in the peripheral blood samples of ASD individuals. It is important to note that Alu elements have the potential to influence gene expression through insertion into the gene structure and through the contribution of gene regulatory elements, such as transcription binding sites and CpG sites [17]. Moreover, Alu elements can act as enhancers, alternate promoters, transcription start sites, and inhibitors of transcription via heterochromatin formation [28, 29].

Several studies have reported altered gene expression profiles in the peripheral blood or blood-derived cell lines of ASD individuals [15, 30–33]. However, no studies have identified an association between DEGs in ASD and Alu elements that could influence gene expression. We first performed multiple gene expression profile comparisons with human Alu-inserted genes in ASD samples using Fisher’s exact test. Interestingly, we found that the Alu-inserted genes were associated with DEGs in ASD individuals in four studies (Table 3). These results indicated a strong association of Alu insertion with downregulated genes in ASD, suggesting that Alu elements could affect gene downregulation. Another interesting question is whether the specific Alu-inserted positions are associated with gene regulation. For this analysis, the Alu-inserted gene list was categorized into four types of Alu insertions: intronic, exonized, exonic, and promoter inserts. Interestingly, we found that intronic Alu insertion was significantly associated with DEGs in ASD invididuals. According to a previous study by Tsirigos et al., a genome-wide computational analysis showed that Alu elements selectively retained in the intronic region of inserted genes were associated with specific functions, including the regulation of transcription, RNA processing and splicing, and translation [34]. However, it remains unclear how intronic Alu insertions can influence the expression of genes associated with ASD-related biological functions, and this topic should be investigated further in future research.

We then explored the biological functions associated with 320 DEGs with Alu insertions that overlapped between the selected studies. These overlapping genes were significantly associated with autism or intellectual disability and ASD co-morbid disorders, including mental retardation and cognitive impairment. It is noteworthy that sex hormone signaling pathways, including estrogen receptor signaling and androgen signaling pathways, were also associated with DEGs containing Alu elements. ASD is biased towards males with a male-to-female ratio of at least 4:1, and there is accumulating evidence showing that sex hormones and related pathways may play an important role in the sex bias of ASD [35–37]. Recent studies have found that high testosterone exposure during pregnancy significantly correlates with the development of ASD, social development, and language development [38–40]. Moreover, the expression levels of estrogen receptor mRNA and protein were found to be reduced in the brain tissues and serum of ASD individuals [41, 42]. Interestingly, one of the DEGs with Alu insertion related to estrogen receptor and androgen signaling is *NCOA1*, which is a gene encoding the nuclear receptor coactivator 1 protein. This coregulatory protein has been reported to interact with estrogen receptor and androgen receptor, which oppositely regulate the transcription of the *RORA* gene encoding the retinoic acid-related (RAR) orphan receptor-alpha (RORA) protein [36]. RORA is a hormone-dependent transcription factor that regulates many genes, including *CYP19A1* (aromatase), an enzyme that converts testosterone to estrogen [37, 43–45]. Our previous study also found that RORA binds NCOA1 and regulates the transcription of the *CYP19A1* gene [36]. Thus, it is possible that Alu elements are associated with the sex bias of ASD by disrupting the androgen receptor/estrogen receptor-mediated regulation of *RORA* and *CYP19A1*. Other interesting pathways that are known to be associated with ASD include neurotrophin/TRK signaling [46], ERK/MAPK signaling [47], axonal guidance signaling [48], CREB signaling in neurons [49], IL-6 signaling [50], and neuroinflammation signaling [51]. Although these biological functions and pathways associated with DEGs in ASD with Alu element insertion have been strongly implicated in ASD, we cannot exclude the possibility that all Alu-inserted genes might be enriched for these functions or pathways, regardless of whether they exhibit differential expression in ASD. The full list of Alu-inserted genes could have been used for IPA analysis as a control to address this question. However, IPA does not allow the use of a gene list larger than 8000 genes. This issue should be investigated in future research studies using other pathway analysis programs.

To date, several DMVs at specific CpG sites or DMRs have been identified in many ASD tissue types, including LCLs [9], whole blood [10], and brain [11]. However, these studies did not cover noncoding regions, including repetitive sequences and retrotransposons. DNA methylation is the major epigenetic mechanism that represses retrotransposons in the human genome, particularly Alu elements due to their relatively high CpG density [18, 52, 53]. Kochanek and colleagues found that DNA methylation at CpG sites within an internal promoter (B box) of Alu elements could inhibit their transcriptional activity [54]. In this study, we also assessed the DNA methylation and expression level of AluS subfamilies that contributed the most Alu element copies and CpG sites in the human genome [18]. COBRA of AluS was designed to measure CpG methylation at the internal promoter, including the B box, in LCLs from ASD patients and sex- and age-matched controls. The LCLs were representative samples from three phenotypic groups based on previous multivariate cluster analyses of ADI-R scores of 1954 individuals with ASD. Our results revealed that the AluS methylation levels and patterns in the LCLs of the combined group of ASD individuals compared with sex- and age-control groups were not significantly different. However, when LCLs were divided into phenotypic subgroups, the AluS methylation patterns in two of the ASD subgroups were significantly different compared with those in the sex- and age-matched controls. In the ASD subgroup with mild symptoms (M), the AluS methylation pattern ^u^C^m^C was increased compared with that in the sex- and age-matched controls. Furthermore, the AluS methylation pattern ^m^C^u^C was reduced in the ASD subgroup with savant skill (S) compared with the sex- and age-matched controls. Although the abovementioned methylation patterns are statistically significant within a specific ASD subtype, it is also worth noting that other AluS methylation patterns were not much altered in ASD in comparison with control groups. One possible explanation is that AluS elements may be dysregulated only at certain sites in the genome, such as in exonic regions. However, the COBRA analysis used in this study does not measure methylation of AluS at specific genomic locations. Thus, it is likely that signals from significant methylation sites were dampened by noise from a large number of Alu elements located in other non-significant sites in the genome.

We then measured the expression of AluS in the LCLs from the subgroups of ASD and found that AluS was over-expressed in ASD subgroup S and downregulated in the ASD subgroup with severe language impairment (L) relative to their respective sex- and age-matched controls. To investigate whether DNA methylation patterns regulated the expression of Alu elements, we analyzed the correlations between AluS methylation and expression levels. A positive correlation between AluS methylation and expression was only observed in ASD subgroup M and in sex- and age-matched controls in which the methylation pattern ^u^C^m^C showed a moderate correlation with AluS expression. It is interesting to note that the methylation pattern ^u^C^m^C was also significantly increased in subgroup M in the COBRA analysis. These findings suggest that AluS expression may be regulated by the AluS methylation pattern ^u^C^m^C. However, such relationships were not found when all ASD individuals were combined or in other ASD subgroups. In ASD subgroup S, the AluS methylation patterns and expression levels were not correlated, although the AluS methylation pattern ^m^C^u^C was significantly reduced, and AluS expression was over-expressed. Moreover, ASD subgroup L showed a significant reduction in AluS expression, but not DNA methylation, indicating that the decrease in AluS expression in ASD subgroup L might result from other epigenetic or gene regulatory mechanisms, such as the disrupted transcription of genes containing an Alu element by altered transcription factor binding at the promoter of the genes but not at the Alu promoter or the other gene regulatory mechanism that have been implicated in LCLs of ASD individuals [55]. These findings suggest that the AluS regulatory mechanism related to methylation might be unique for a specific ASD subpopulation, and there might be other regulatory mechanisms involved in the regulation of AluS methylation and expression, which should be investigated in future research.

**Fig. 8:**
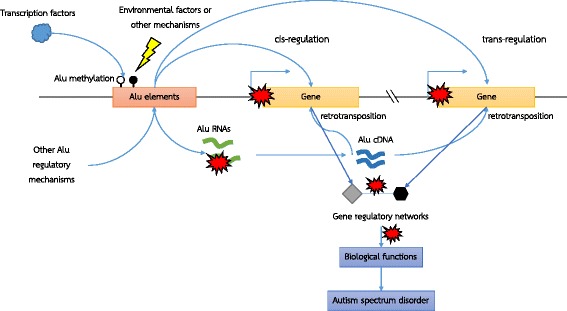
Schematic diagram illustrating a possible mechanism of Alu elements in ASD. Our model suggests that exposure to environmental factors or dysregulation of other DNA methylation regulatory mechanisms lead to changes in CpG methylation patterns in Alu elements. Such changes alter transcription factor binding and, possibly in combination with other Alu regulatory mechanisms, cause the dysregulation of the expression and retrotransposition of Alu elements. Disrupted Alu retrotransposition results in changes in target genes via cis-/trans-regulatory mechanisms, which, in turn, dysregulate gene expression and gene regulatory networks known to be negatively impacted in ASD

The changes of Alu RNAs in LCLs from some ASD subgroups might reflect molecular function because Alu RNAs are involved in transcriptome diversity by contributing recognition sites for RNA editing and alternative splicing within Alu sequences [56]. Altered DNA methylation within Alu elements may have a negative impact on gene regulatory networks, which in turn, may affect the biological functions associated with ASD (Fig. 8). The exact mechanisms remain unclear, but it is possible that altered methylation within the Alu promoter, together with other gene regulatory mechanisms, may lead to changes in Alu transcript expression and retrotransposition. Because Alu elements contain transcription factor binding sites within their sequences, these elements could disrupt gene structure and functions by serving as an alternative transcription start site, enhancer, or promoter for cis-/trans-regulation of the target genes once inserted into a new genomic location by the retrotransposition [28, 29, 57–59].

We obtained transcriptomic data from five different studies that used different sample types (e.g., whole blood, leukocytes, and LCLs) as well as different cohorts of subjects with ASD. It remains unclear whether ASD gene expression or methylation signatures will be similar across tissue sources. There might be variations in gene expression and methylation patterns among different sample types and among different cells in the same tissue type. This issue is a potential limitation of this study and should be investigated further in future research. In addition, some of the five selected transcriptome studies did not subgroup ASD individuals before performing DEG analysis and there were no ADI-R scores available for meta-analysis, whereas others used different criteria and strategies to divide ASD into subgroups. It is therefore difficult to relate our methylation analysis findings to previous analyses of Alu-inserted DEGs in which all ASD individuals were combined or studies using different subgrouping strategies. However, we performed Fisher’s exact tests using the microarray data from a previous study (GSE15402) that used the same subgrouping criteria and the same cell model (LCLs) used in this study. Interestingly, we found that genes with Alu element insertion were associated with specific subgroups of ASD rather than with all individuals with ASD. The results of Alu methylation analysis using COBRA and Alu expression analysis using qRT-PCR also showed that the dysregulation of Alu methylation and Alu expression were observed only when ASD cases were divided into subgroups. This finding suggests that the subgrouping of ASD individuals will help reduce heterogeneity and may lead to the discovery of novel mechanisms associated with Alu element in ASD subpopulations. A better understanding of the molecular mechanisms specific for each ASD subgroup might allow the identification of biomarkers and treatment strategies personalized to each subgroup in the future. However, the sample size of this study is relatively small, especially when individuals with ASD were further divided into subgroups. The role of Alu in the context of ASD deserves further studies using a larger cohort. Most importantly, more experiments are needed to verify that Alu elements play important roles in ASD. Brain tissue samples may be used to confirm these findings, and single-cell RNA sequencing might help reduce cell heterogeneity.

## Conclusion

Our findings show that the DEGs in males with ASD from several studies with different sample groups are associated with human Alu elements. Differentially expressed genes with Alu insertions were associated with neurodevelopmental disorders and neurological functions involved in the etiology of ASD. In particular, genes involved in estrogen receptor and androgen signaling pathways, which have been reported to be related with sex bias in ASD, were identified as DEGs with Alu insertions. In addition, the global methylation of AluS subfamilies in LCLs was investigated and revealed different AluS methylation patterns within specific ASD phenotypic subgroups. Our findings suggest that the classification of ASD patients into subgroups based on the clinical or behavioral phenotypes of individual patients might help improve our understanding of ASD etiology by reducing the inherent heterogeneity within the ASD population. In addition, this study provides suggestive evidence for an association between Alu elements and ASD.

## Additional files

Additional file 1: Demographic information of LCLs used in this study. (DOC 73 kb)

Additional file 2: Association analysis between the DEGs in ASD from GSE15402 and the human Alu-inserted genes lists when ASD individuals were sub-grouped based on ADI-R scores. The comparisons were performed for five types of Alu insertions, including exonized, exonic, intronic, promoter and all types. The Fisher’s exact test *P* value and number of DEGs are shown. (DOC 46 kb)

Additional file 3: List of the overlapping genes. (DOC 118 kb)

Additional file 4: Levels and patterns of Alu methylation in each individual. (DOC 98 kb)

Additional file 5: Correlation analysis between AluS methylation and expression level of ASD subgroup L. (PDF 329 kb)

Additional file 6: Correlation analysis between AluS methylation and expression level of ASD subgroup S. (PDF 333 kb)

## Abbreviations

ADI-R: Autism Diagnostic Interview-Revised
ASD: Autism spectrum disorder
COBRA: Combined bisulfite restriction analysis
DEGs: Differentially expressed genes
DMRs: Differentially methylated regions
DMVs: Differentially methylated variants
DSM-5: The Diagnostic and Statistical Manual of Mental Disorders, Fifth Edition
ERK: Extracellular signal-regulated kinases
GEO: Gene Expression Omnibus
IPA: Ingenuity Pathway Analysis
LCLs: Lymphoblastoid cell lines
MAPK: Mitogen-activated protein kinases
RORA: Retinoic acid receptor-related orphan receptor alpha
RPMI: Roswell Park Memorial Institute
RT-PCR: Reverse transcription-polymerase chain reaction
SINEs: Short interspersed elements
TEs: Transposed elements
TRK: Tyrosine kinases
