# Supplementary material for: Integrated genome-wide Alu methylation and transcriptome profiling analyses reveal novel epigenetic regulatory networks associated with autism spectrum disorder
Source: Mol Autism. 2018 Apr 16;9:27. doi: 10.1186/s13229-018-0213-9 (PMC5902935; doi:10.1186/s13229-018-0213-9)
Supplement: Supplementary file 1 — Demographic information of LCLs used in this study. (DOC 73 kb) [file 13229_2018_213_MOESM1_ESM.doc]

**Additional file 1. Demographic information of LCLs used in this study.**

| **Individual Code** | **BloodID** | **Age** | **AGRE (ADIR) diagnosis** | **Ethnicity** |
| --- | --- | --- | --- | --- |
| AU0955303 | HI2791 | 6 | Autism | Hispanic or Latino |
| AU1102303 | HI2824 | 7 | Autism | Hispanic or Latino |
| AU005215 | HI1276 | 9 | Autism | Not Hispanic or Latino |
| AU083604 | HI2163 | 9 | Autism | Not Hispanic or Latino |
| AU1164302 | HI2883 | 9 | Autism | Not Hispanic or Latino |
| AU069603 | HI1429 | 11 | Autism | Not Hispanic or Latino |
| AU069604 | HI1428 | 11 | Autism | Not Hispanic or Latino |
| AU029803 | HI0624 | 13 | Autism | Not Hispanic or Latino |
| AU043604 | HI0700 | 13 | Autism | Not Hispanic or Latino |
| AU1048302 | HI2677 | 13 | Autism | Hispanic or Latino |
| AU1102301 | HI2815 | 13 | Autism | Hispanic or Latino |
| AU080403 | HI2039 | 14 | Autism | Not Hispanic or Latino |
| AU060004 | HI0927 | 14 | Autism | Not Hispanic or Latino |
| AU048103 | HI1555 | 15 | Autism | Not Hispanic or Latino |
| AU1048301 | HI2679 | 16 | Autism | Hispanic or Latino |
| AU067703 | HI2008 | 18 | Autism | Not Hispanic or Latino |
| AU005303 | HI1102 | 18 | Autism | Not Hispanic or Latino |
| AU070808 | HI1911 | 21 | Autism | Not Hispanic or Latino |
| AU005604 | HI0613 | 28 | Autism | Not Hispanic or Latino |
| AU1546302 | HI4341 | 7 | Autism | Hispanic or Latino |
| AU1648301 | HI4751 | 9 | Autism | Not Hispanic or Latino |
| AU056604 | HI1234 | 10 | Autism | Not Hispanic or Latino |
| AU1196301 | HI4870 | 15 | Autism | Not Hispanic or Latino |
| AU081203 | HI2028 | 16 | Autism | Not Hispanic or Latino |
| AU1008302 | HI4461 | 16 | Autism | Hispanic or Latino |
| AU016803 | HI1492 | 17 | Autism | Not Hispanic or Latino |
| AU015003 | HI0591 | 12 | Autism | Not Hispanic or Latino |
| AU062203 | HI1943 | 12 | Autism | Not Hispanic or Latino |
| AU051504 | HI0792 | 13 | Autism | Not Hispanic or Latino |
| AU079103 | HI1861 | 13 | Autism | Unknown |
| AU041904 | HI0649 | 15 | Autism | Not Hispanic or Latino |
| AU015903 | HI0928 | 17 | Autism | Not Hispanic or Latino |
| AU043203 | HI0652 | 22 | BroadSpectrum | Not Hispanic or Latino |
| AU1685302 | HI4838 | 8 | BroadSpectrum | Not Hispanic or Latino |
| AU073804 | HI2251 | 13 | BroadSpectrum | Not Hispanic or Latino |
| AU053504 | HI1495 | 10 | NQA | Not Hispanic or Latino |
| AU069606 | HI4281 | 3 | Unaffected | Not Hispanic or Latino |
| AU083605 | HI2162 | 7 | Unaffected | Not Hispanic or Latino |
| AU1429304 | HI4090 | 8 | Unaffected | Not Hispanic or Latino |
| AU059407 | HI1534 | 9 | Unaffected | Not Hispanic or Latino |
| AU007505 | HI0742 | 10 | Unaffected | Not Hispanic or Latino |
| AU1135203 | HI2725 | 10 | Unaffected | Hispanic or Latino |
| AU045012 | HI1866 | 11 | Unaffected | Not Hispanic or Latino |
| AU020105 | HI0507 | 13 | Unaffected | Not Hispanic or Latino |
| AU059406 | HI1537 | 13 | Unaffected | Not Hispanic or Latino |
| AU059405 | HI1535 | 14 | Unaffected | Not Hispanic or Latino |
| AU061405 | HI1545 | 14 | Unaffected | Not Hispanic or Latino |
| AU057903 | HI0813 | 16 | Unaffected | Not Hispanic or Latino |
| AU053304 | HI1788 | 16 | Unaffected | Not Hispanic or Latino |
| AU059403 | HI1539 | 18 | Unaffected | Not Hispanic or Latino |
| AU032503 | HI0365 | 20 | Unaffected | Unknown |
| AU019404 | HI1047 | 20 | Unaffected | Not Hispanic or Latino |
| AU077503 | HI1706 | 20 | Unaffected | Not Hispanic or Latino |
| AU0885304 | HI2357 | 20 | Unaffected | Unknown |
| AU0885301 | HI2356 | 24 | Unaffected | Unknown |
| AU062903 | HI1161 | 34 | Unaffected | Not Hispanic or Latino |
