# Supplementary material for: Integrated genome-wide Alu methylation and transcriptome profiling analyses reveal novel epigenetic regulatory networks associated with autism spectrum disorder
Source: Mol Autism. 2018 Apr 16;9:27. doi: 10.1186/s13229-018-0213-9 (PMC5902935; doi:10.1186/s13229-018-0213-9)
Supplement: Supplementary file 2 — Association analysis between the DEGs in ASD from GSE15402 and the human Alu-inserted genes lists when ASD individuals were sub-grouped based on ADI-R scores. The comparisons were performed for five types of Alu insertions, including exonized, exonic, intronic, promoter and all types. The Fisher’s exact test P value and number of DEGs are shown. (DOC 46 kb) [file 13229_2018_213_MOESM2_ESM.doc]

**Additional file 2.** **Association analysis between the DEGs in ASD from GSE15402 and the human Alu-inserted genes lists, when ASD individuals were sub-grouped based on ADI-R scores.**

| **Insertion type** | **Comparison** | **Differentially expressed genes** | | **Up-regulated genes** | | **Down-regulated genes** | |
| --- | --- | --- | --- | --- | --- | --- | --- |
|  |  | **P-value** | **Genes(n)** | **P-value** | **Genes(n)** | **P-value** | **Genes(n)** |
| All insertion | ASD *vs* C | 1.000 | 215 | 1.000 | 100 | 1.000 | 116 |
|  | M *vs* C | 1.000 | 311 | 1.000 | 202 | 1.000 | 111 |
|  | L *vs* C | 0.395 | 837 | 1.000 | 379 | 0.033 | 465 |
|  | S *vs* C | 1.000 | 140 | 1.000 | 74 | 1.000 | 66 |
| Intronic | ASD *vs* C | 1.000 | 212 | 1.000 | 99 | 1.000 | 114 |
|  | M *vs* C | 1.000 | 309 | 1.000 | 202 | 1.000 | 109 |
|  | L *vs* C | 0.537 | 823 | 1.000 | 372 | 0.033 | 458 |
|  | S *vs* C | 1.000 | 138 | 1.000 | 73 | 1.000 | 65 |
| Exonized | ASD *vs* C | 1.000 | 15 | 1.000 | 6 | 1.000 | 9 |
|  | M *vs* C | 0.0735 | 35 | 0.0735 | 25 | 1.000 | 11 |
|  | L *vs* C | 0.4340 | 72 | 1.000 | 34 | 0.762 | 39 |
|  | S *vs* C | 1.000 | 10 | 1.000 | 5 | 1.000 | 5 |
| Exonic | ASD *vs* C | 1.000 | 19 | 1.000 | 10 | 1.000 | 9 |
|  | M *vs* C | 1.000 | 32 | 1.000 | 17 | 1.000 | 15 |
|  | L *vs* C | 1.000 | 102 | 1.000 | 48 | 1.000 | 54 |
|  | S *vs* C | 1.000 | 14 | 1.000 | 8 | 1.000 | 6 |
| Promoter | ASD *vs* C | 1.000 | 6 | 1.000 | 1 | 1.000 | 5 |
|  | M *vs* C | 1.000 | 11 | 1.000 | 8 | 1.000 | 3 |
|  | L *vs* C | 1.000 | 26 | 1.000 | 12 | 1.000 | 14 |
|  | S *vs* C | 1.000 | 4 | 1.000 | 4 | 1.000 | 0 |
