# Supplementary material for: Integrated genome-wide Alu methylation and transcriptome profiling analyses reveal novel epigenetic regulatory networks associated with autism spectrum disorder
Source: Mol Autism. 2018 Apr 16;9:27. doi: 10.1186/s13229-018-0213-9 (PMC5902935; doi:10.1186/s13229-018-0213-9)
Supplement: Supplementary file 3 — List of the overlapping genes. (DOC 118 kb) [file 13229_2018_213_MOESM3_ESM.doc]

**Additional file 3. List of the overlapping genes**

| **Overlapping studies** | **Number** | **Overlapping genes** | | | |
| --- | --- | --- | --- | --- | --- |
| GSE18123 vs GSE25507 vs GSE42133 | 8 | BBX | UBE4B | PTPRE | WNK1 |
|  |  | SENP5 | PHF20L1 | JARID2 | PAK2 |
| GSE18123 vs GSE42133 vs GSE6575 | 9 | AHNAK | YY1 | MBP | TRRAP |
|  |  | SRRM1 | HSPA5 | GNB1 | AP3D1 |
|  |  | ABCB1 |  |  |  |
| GSE18123 vs GSE25507 vs GSE6575 | 2 | CDS2 | ZNF207 |  |  |
| GSE25507 vs GSE42133 vs GSE6575 | 1 | CASP2 |  |  |  |
| GSE18123 vs GSE42133 | 79 | MYO9B | HGF | TWSG1 | SAP130 |
|  |  | SEMA4D | ARID1A | NAP1L1 | UBL3 |
|  |  | WHSC1L1 | ITGAL | PTPRC | PIK3CG |
|  |  | PPM1A | IQGAP2 | PPP3CB | MBNL1 |
|  |  | SON | TOMM20 | WDR7 | SSH1 |
|  |  | PLCL2 | CLN8 | OSBPL1A | DIAPH1 |
|  |  | APPBP2 | PTPN11 | FER | STK4 |
|  |  | RASSF5 | MYH9 | MPP5 | CA2 |
|  |  | BBS1 | MBTPS1 | PAN3 | IL6ST |
|  |  | CAMSAP1 | RCOR3 | SSR1 | ELF2 |
|  |  | SERTAD2 | ZNF540 | ZNF559 | PTPRA |
|  |  | RDX | FCER1A | PURB | MGAT4A |
|  |  | SVIL | WDR4 | PIK3R1 | DSCR3 |
|  |  | TLK1 | KIAA2026 | HOOK1 | IL7R |
|  |  | CREBBP | TAF4 | TCF20 | UBQLN1 |
|  |  | NACA | SPIN1 | UBE3A | CANX |
|  |  | IRS2 | ETF1 | IFNAR2 | SSX2IP |
|  |  | LIMS1 | ZCCHC14 | PDK3 | SMARCA2 |
|  |  | YES1 | PKN2 | AAK1 | PGLS |
|  |  | WWP1 | PIK3R4 | ZNF12 |  |
| GSE18123 vs GSE25507 | 144 | PELI2 | MAP2K4 | CFLAR | VPS13D |
|  |  | TRIM33 | WDR26 | TBRG1 | PLAA |
|  |  | OSBPL8 | GSK3B | ADNP | PGM2 |
|  |  | TNS3 | KIF1B | NSMAF | PECAM1 |
|  |  | TSC22D1 | PIAS1 | UPF2 | LYN |
|  |  | FPGT | NCOA6 | APC | DGKG |
|  |  | EIF1AX | SPAST | TLR2 | SNX27 |
|  |  | ALCAM | TRIP11 | PTEN | SLC16A6 |
|  |  | SLC6A6 | HMGCR | TRIO | PIGA |
|  |  | QKI | UBR1 | VCPIP1 | CBL |
|  |  | TMEM33 | ZCCHC6 | TRIM23 | ZNF451 |
|  |  | ARIH1 | ABHD5 | MAP3K5 | STX12 |
|  |  | WDR20 | USP15 | TBK1 | NIN |
|  |  | CNOT6 | ZNF493 | DICER1 | SRPK2 |
|  |  | NFYA | MAPK1 | CNOT4 | SPG21 |
|  |  | NFE2L2 | PPP3R1 | LPGAT1 | ANKFY1 |
|  |  | OPA1 | PAPOLG | MOSPD2 | ENTPD4 |
|  |  | IDS | CCPG1 | SLC15A4 | DDX6 |
|  |  | ASXL2 | BAZ2B | ANTXR2 | KLHL8 |
|  |  | YPEL5 | TNFSF8 | PLXNC1 | SLC20A1 |
|  |  | ITGB1 | TXNRD1 | STK38L | MTRF1L |
|  |  | MAP1LC3B | USP33 | LYST | APLP2 |
|  |  | SPAG9 | CYBB | USP25 | MSI2 |
|  |  | CORO1C | NCOA1 | CNNM4 | TGOLN2 |
|  |  | HBP1 | RAB22A | ADAM10 | RIT1 |
|  |  | NF1 | RHOQ | CDC42 | ZNF180 |
|  |  | FAM49A | SHOC2 | COL4A3BP | ARID4A |
|  |  | ATP6V1A | KIF13A | TRERF1 | ANKRD12 |
|  |  | ANKRD17 | DDX17 | AK2 | BACH1 |
|  |  | NOTCH2 | NAB1 | PRKAG2 | DOCK5 |
|  |  | RRAGC | NHS | UBE2B | SLC35A3 |
|  |  | TMOD2 | TAB3 | ELMOD2 | NOV |
|  |  | ARHGAP26 | LAMP2 | CSNK1A1 | RNF130 |
|  |  | PAPD4 | ATXN1 | RAB31 | PTP4A1 |
|  |  | ZAK | TMF1 | TOP1 | UBR2 |
|  |  | FAM49B | HIPK1 | KPNB1 | MAP4K4 |
| GSE18123 vs GSE6575 | 27 | ELMO2 | MYO5A | DOCK8 | AP1G1 |
|  |  | GLG1 | TNPO3 | XRCC6 | DOCK2 |
|  |  | FYN | ETS1 | PSMB2 | GIMAP8 |
|  |  | LDLR | CHD4 | TPCN2 | TGFBI |
|  |  | SAMHD1 | ANAPC5 | ANKRD11 | NPC1 |
|  |  | TFCP2 | EWSR1 | ZNF652 | XPO5 |
|  |  | GCN1L1 | CTBP2 | ATP2A2 |  |
| GSE25507 vs GSE42133 | 12 | RAB3IP | XPR1 | CREB1 | MYCBP |
|  |  | SHC1 | SP1 | GNAI2 | PPP2R5E |
|  |  | RAD23B | CPNE3 | USP13 | CNOT7 |
| GSE42133 vs GSE6575 | 33 | GLTP | WDR1 | INO80 | SART3 |
|  |  | PROSC | SMPD1 | DRG2 | PABPC1 |
|  |  | RUNX3 | GTF3C2 | FNBP1 | PSMD7 |
|  |  | DSTN | PRKCSH | LGR6 | P4HB |
|  |  | WBP11 | TBL1X | DDX27 | AKAP8 |
|  |  | SAFB2 | MEN1 | PEX14 | ANXA7 |
|  |  | TDP1 | PRPF4 | KIF13B | ARCN1 |
|  |  | BUB3 | S100A10 | ZMYND11 | SLC35E1 |
|  |  | GORASP2 |  |  |  |
| GSE25507 vs GSE6575 | 5 | QDPR | CAMTA1 | EHD4 | SYNCRIP |
|  |  | PSME3 |  |  |  |
