# Supplementary material for: Integrated genome-wide Alu methylation and transcriptome profiling analyses reveal novel epigenetic regulatory networks associated with autism spectrum disorder
Source: Mol Autism. 2018 Apr 16;9:27. doi: 10.1186/s13229-018-0213-9 (PMC5902935; doi:10.1186/s13229-018-0213-9)
Supplement: Supplementary file 4 — Levels and patterns of Alu methylation in each individual. (DOC 98 kb) [file 13229_2018_213_MOESM4_ESM.doc]

**Additional file 4. Levels and patterns of Alu methylation in each individual.**

| **Groups** | **Sample** | **%^m^C** | **%^m^C^m^C** | **%^u^C^m^C** | **%^m^C^u^C** | **%^u^C^u^C** |
| --- | --- | --- | --- | --- | --- | --- |
| Control | C0365 | 37.44 | 25.52 | 17.63 | 22.18 | 34.67 |
| Control | C0507 | 37.55 | 22.98 | 21.09 | 22.42 | 33.52 |
| Control | C0742 | 37.29 | 24.23 | 18.83 | 22.48 | 34.46 |
| Control | C0813 | 40.05 | 27.91 | 18.52 | 22.48 | 31.08 |
| Control | C1047 | 39.65 | 28.13 | 18.91 | 21.11 | 31.85 |
| Control | C1161 | 36.62 | 25.86 | 18.10 | 19.81 | 36.23 |
| Control | C1534 | 38.72 | 26.53 | 18.64 | 22.01 | 32.83 |
| Control | C1535 | 36.39 | 22.68 | 18.89 | 22.97 | 35.47 |
| Control | C1537 | 38.80 | 25.45 | 20.27 | 21.99 | 32.28 |
| Control | C1539 | 36.92 | 25.91 | 16.93 | 21.42 | 35.73 |
| Control | C1545 | 38.21 | 28.68 | 16.45 | 20.34 | 34.53 |
| Control | C1706 | 35.86 | 22.27 | 18.66 | 22.81 | 36.26 |
| Control | C1788 | 38.01 | 25.01 | 19.28 | 22.23 | 33.49 |
| Control | C1866 | 36.01 | 21.59 | 19.38 | 23.28 | 35.75 |
| Control | C2162 | 38.11 | 25.58 | 19.38 | 21.50 | 33.53 |
| Control | C2354 | 37.04 | 23.44 | 18.96 | 23.01 | 34.59 |
| Control | C2357 | 38.57 | 25.88 | 20.64 | 20.65 | 32.83 |
| Control | C2725 | 37.76 | 27.52 | 17.59 | 20.02 | 34.87 |
| Control | C4090 | 40.57 | 30.07 | 18.15 | 20.71 | 31.06 |
| Control | C4281 | 40.02 | 28.81 | 17.93 | 21.77 | 31.49 |
| Subgroup M | M1234 | 37.24 | 25.03 | 18.67 | 21.45 | 34.85 |
| Subgroup M | M1492 | 37.84 | 23.26 | 20.11 | 23.51 | 33.12 |
| Subgroup M | M1495 | 39.72 | 23.98 | 21.94 | 24.00 | 30.08 |
| Subgroup M | M2028 | 40.72 | 26.37 | 20.14 | 24.19 | 29.30 |
| Subgroup M | M2251 | 39.31 | 26.09 | 19.57 | 22.70 | 31.64 |
| Subgroup M | M4344 | 38.80 | 25.63 | 19.91 | 22.12 | 32.34 |
| Subgroup M | M4461 | 38.44 | 24.29 | 20.16 | 23.08 | 32.46 |
| Subgroup M | M4751 | 38.78 | 24.80 | 20.19 | 22.95 | 32.05 |
| Subgroup M | M4838 | 38.57 | 25.19 | 19.00 | 23.23 | 32.58 |
| Subgroup M | M4870 | 40.87 | 27.03 | 20.95 | 22.70 | 29.32 |
| Subgroup L | L0591 | 36.59 | 25.07 | 18.21 | 20.72 | 35.99 |
| Subgroup L | L0649 | 37.07 | 25.07 | 17.76 | 22.01 | 35.16 |
| Subgroup L | L0792 | 34.58 | 25.40 | 19.02 | 15.97 | 39.61 |
| Subgroup L | L0928 | 36.66 | 20.65 | 22.41 | 22.70 | 34.24 |
| Subgroup L | L1861 | 37.35 | 24.61 | 18.58 | 22.32 | 34.49 |
| Subgroup L | L1943 | 33.88 | 19.41 | 19.93 | 21.85 | 38.81 |
| Subgroup S | S0613 | 40.12 | 28.85 | 20.13 | 19.68 | 31.34 |
| Subgroup S | S0624 | 34.23 | 21.21 | 19.04 | 20.94 | 38.80 |
| Subgroup S | S0652 | 38.33 | 28.25 | 15.84 | 21.75 | 34.16 |
| Subgroup S | S0700 | 41.21 | 31.72 | 17.41 | 20.23 | 30.64 |
| Subgroup S | S0927 | 40.32 | 29.46 | 18.39 | 20.91 | 31.24 |
| Subgroup S | S1102 | 40.92 | 30.42 | 19.95 | 19.01 | 30.62 |
| Subgroup S | S1276 | 35.28 | 24.17 | 17.92 | 19.95 | 37.97 |
| Subgroup S | S1428 | 39.60 | 27.46 | 18.30 | 22.57 | 31.67 |
| Subgroup S | S1429 | 35.41 | 25.77 | 16.96 | 18.95 | 38.31 |
| Subgroup S | S1555/3 | 36.89 | 24.04 | 19.82 | 21.06 | 35.08 |
| Subgroup S | S1911 | 38.42 | 28.05 | 17.97 | 20.04 | 33.94 |
| Subgroup S | S2008 | 35.03 | 27.36 | 14.90 | 18.20 | 39.53 |
| Subgroup S | S2039 | 39.73 | 29.30 | 17.60 | 20.93 | 32.17 |
| Subgroup S | S2163 | 40.73 | 30.56 | 17.20 | 21.26 | 30.99 |
| Subgroup S | S2677 | 37.27 | 27.38 | 15.32 | 21.64 | 35.66 |
| Subgroup S | S2679 | 36.61 | 23.96 | 19.30 | 21.20 | 35.54 |
| Subgroup S | S2791 | 37.71 | 26.14 | 18.75 | 20.67 | 34.44 |
| Subgroup S | S2815 | 36.30 | 26.77 | 17.84 | 18.28 | 37.12 |
| Subgroup S | S2824 | 37.28 | 26.38 | 17.10 | 21.24 | 35.27 |
| Subgroup S | S2883 | 35.11 | 23.98 | 17.77 | 20.05 | 38.20 |
