# Supplementary figures and images for: Integrated genome-wide Alu methylation and transcriptome profiling analyses reveal novel epigenetic regulatory networks associated with autism spectrum disorder
Source: Mol Autism. 2018 Apr 16;9:27. doi: 10.1186/s13229-018-0213-9 (PMC5902935; doi:10.1186/s13229-018-0213-9)

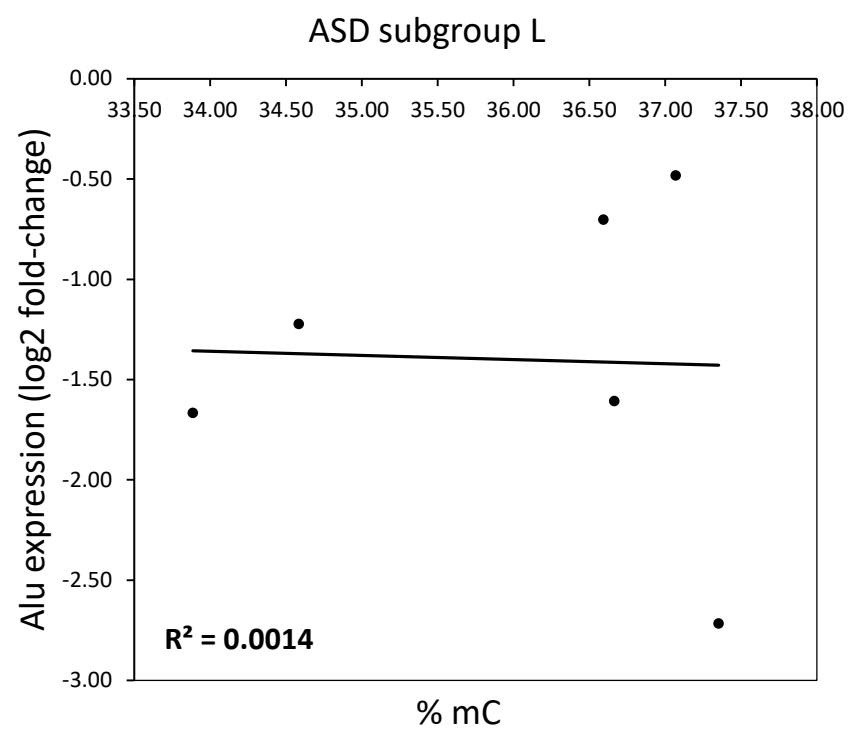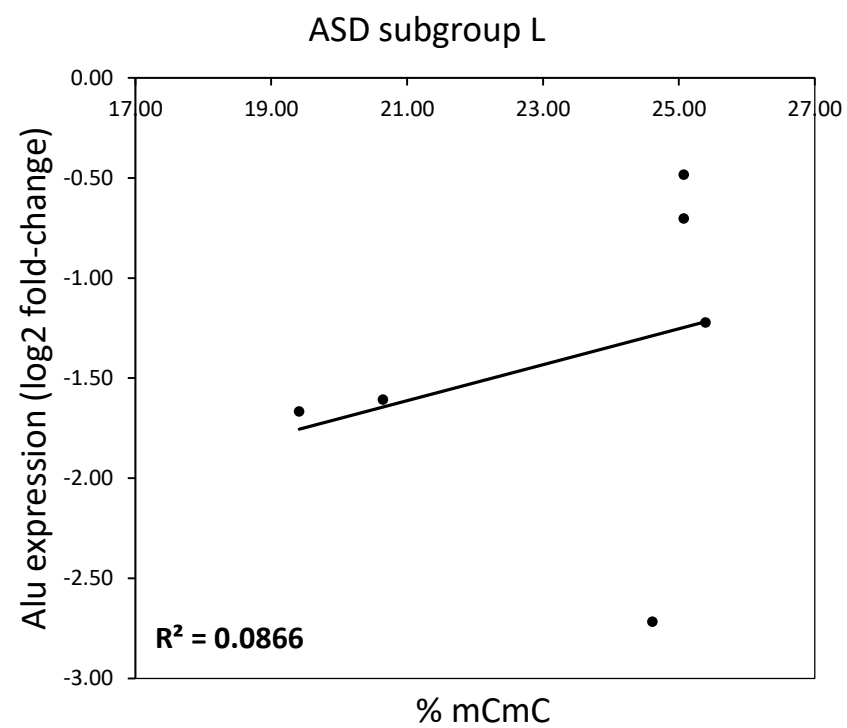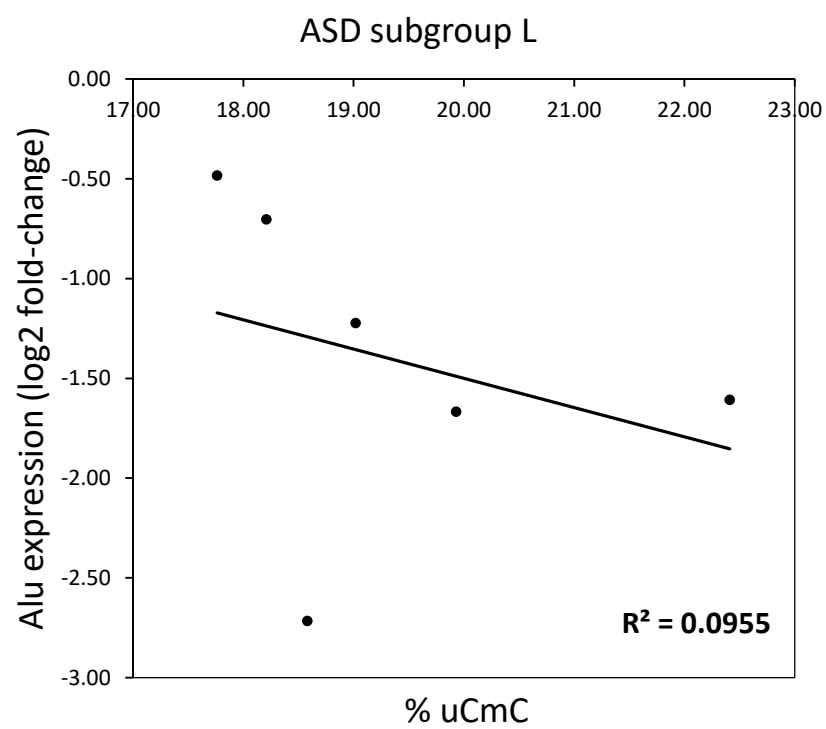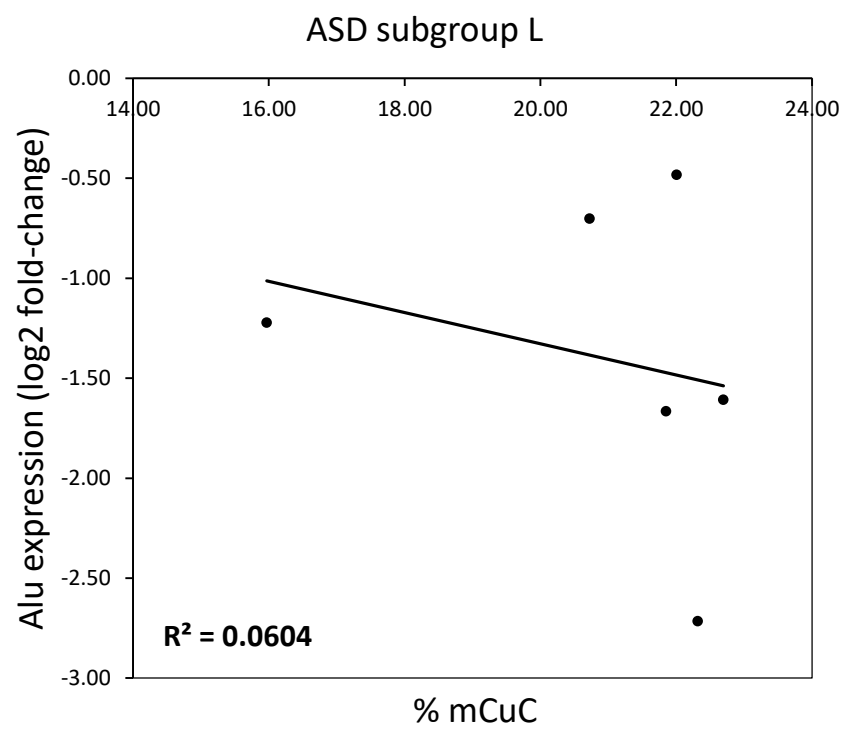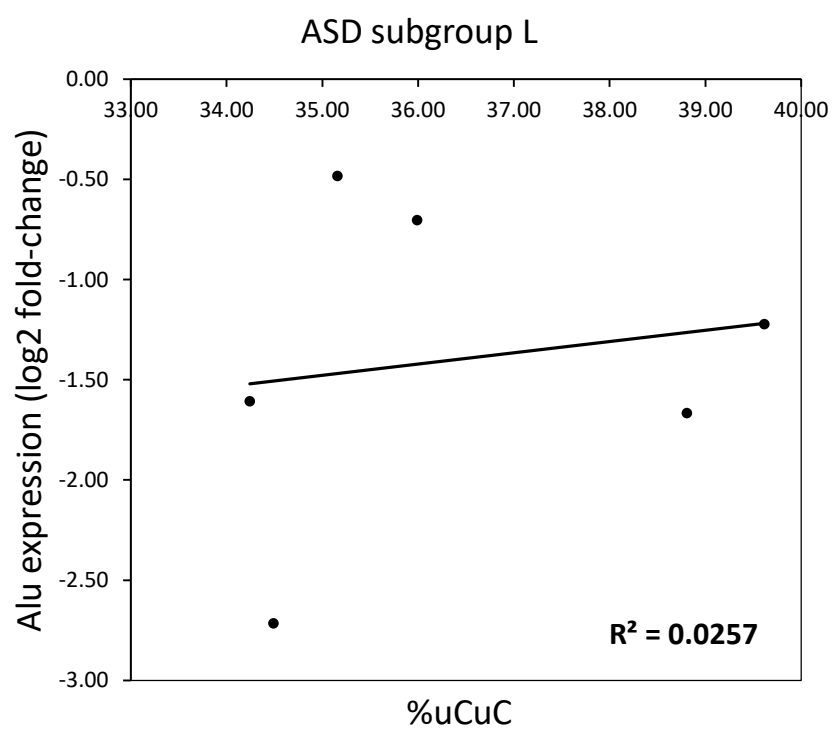

Supplement: Supplementary file 5 — Correlation analysis between AluS methylation and expression level of ASD subgroup L. (PDF 329 kb) [file 13229_2018_213_MOESM5_ESM.pdf]

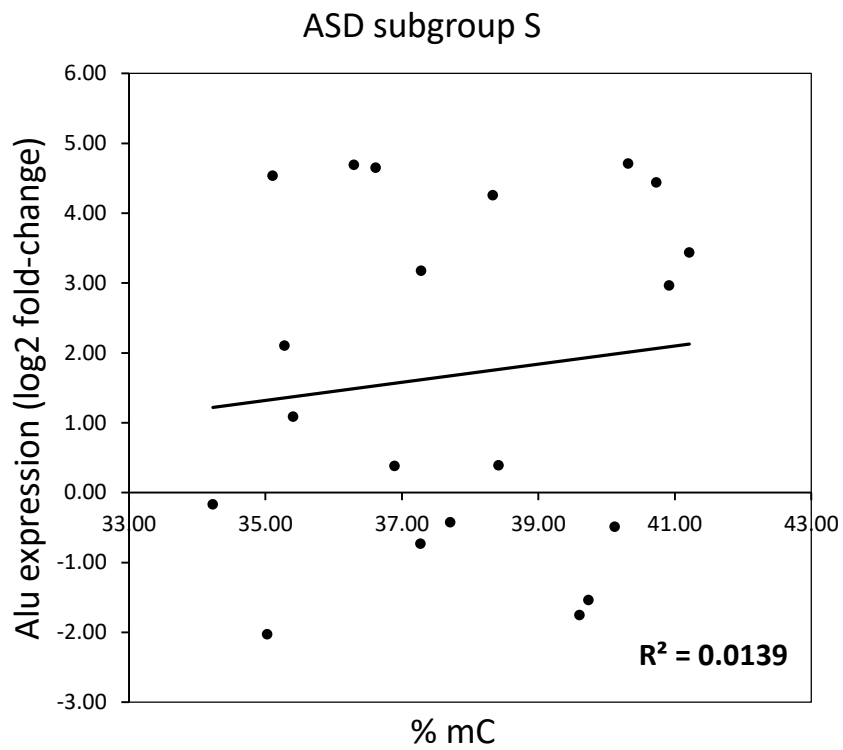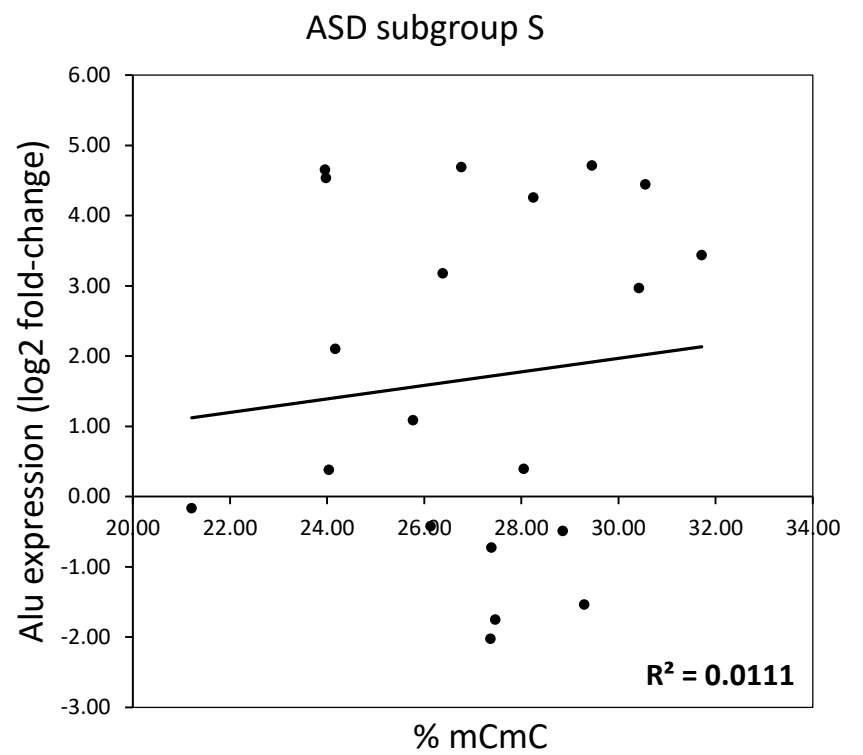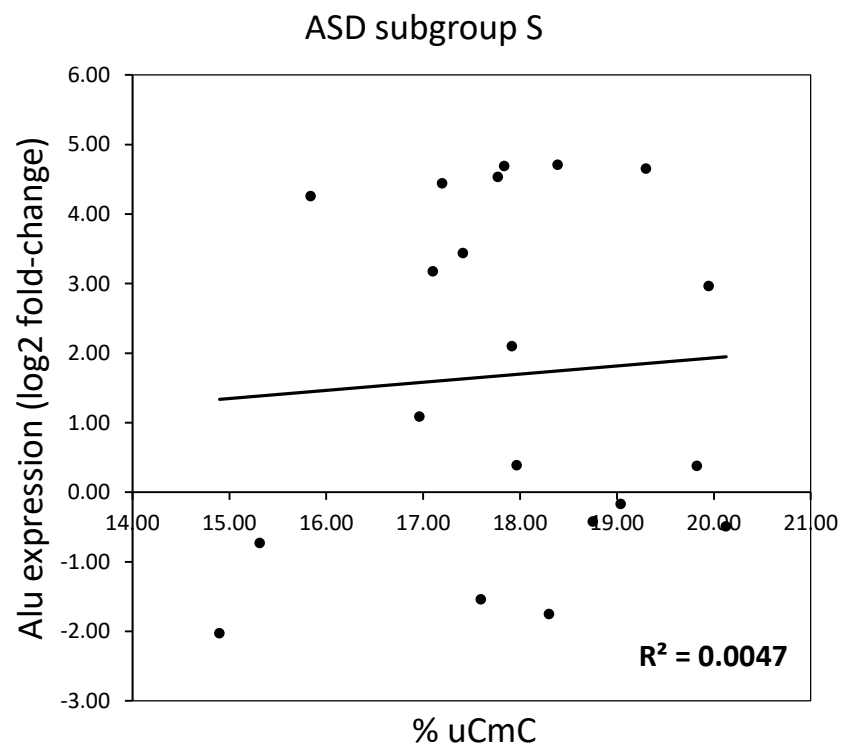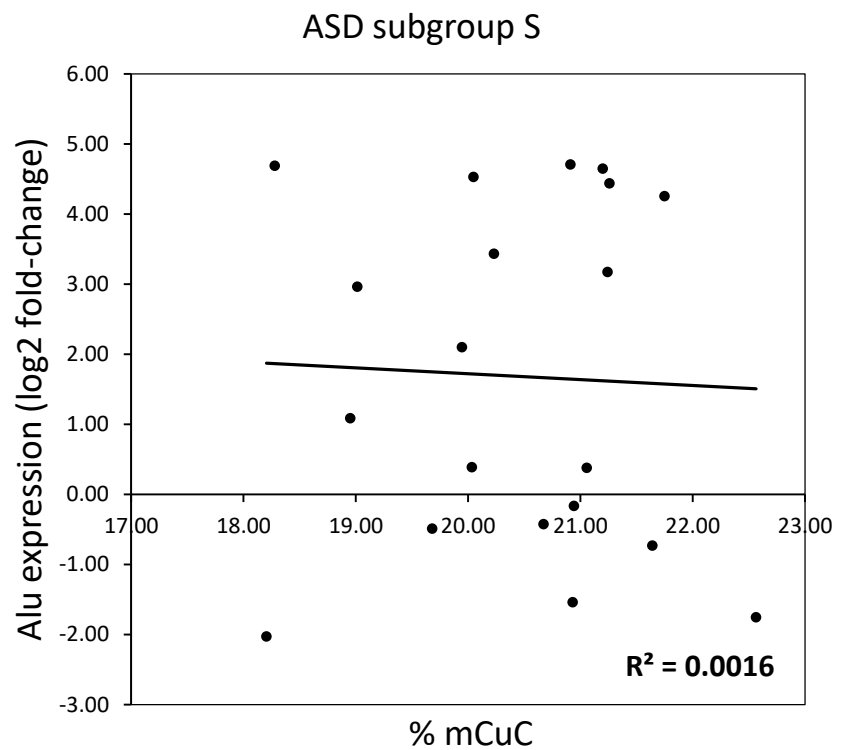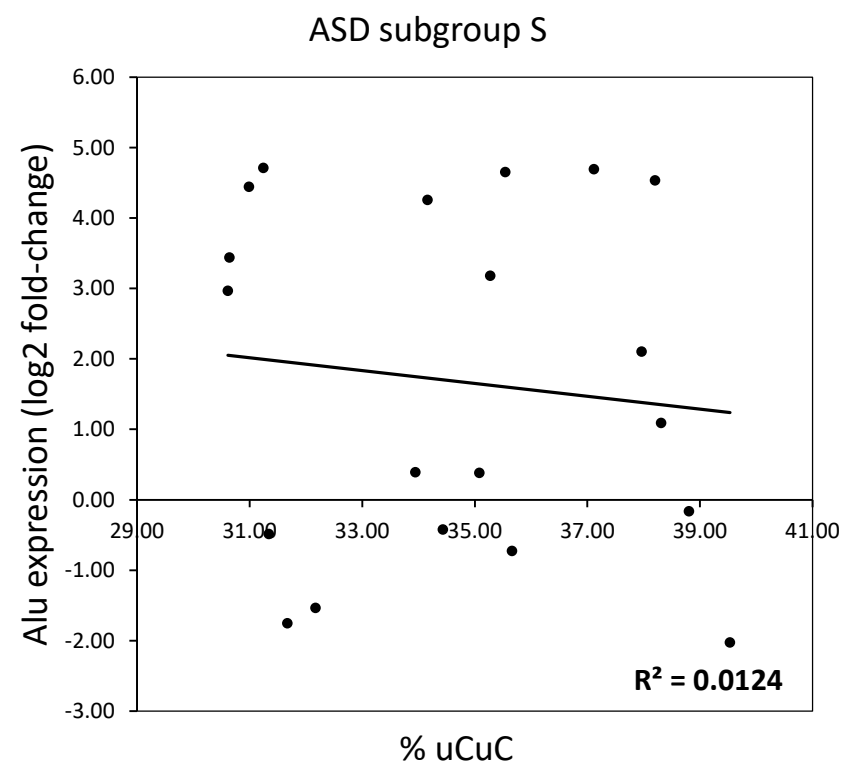

Supplement: Supplementary file 6 — Correlation analysis between AluS methylation and expression level of ASD subgroup S. (PDF 333 kb) [file 13229_2018_213_MOESM6_ESM.pdf]
